# SARS-CoV-2 introductions to the island of Ireland: a phylogenetic and geospatiotemporal study of infection dynamics

**DOI:** 10.1186/s13073-024-01409-1

**Published:** 2024-12-19

**Authors:** Alan M. Rice, Evan P. Troendle, Stephen J. Bridgett, Behnam Firoozi Nejad, Jennifer M. McKinley, The COVID-19 Genomics UK consortium, National SARS-CoV-2 Surveillance & Whole Genome Sequencing (WGS) Programme, Declan T. Bradley, Derek J. Fairley, Connor G. G. Bamford, Timofey Skvortsov, David A. Simpson

**Affiliations:** 1 Wellcome-Wolfson Institute for Experimental Medicine, School of Medicine, Dentistry and Biomedical Sciences, Queen’s University Belfast, Belfast, Northern Ireland, BT9 7BL UK; 2 Geography, School of Natural and Built Environment, Queen’s University Belfast, Belfast, Northern Ireland, BT7 1NN UK; 3 Public Health Agency, Belfast, Northern Ireland, BT2 8BS UK; 4 Centre for Public Health, School of Medicine, Dentistry and Biomedical Sciences, Queen’s University Belfast, Belfast, Northern Ireland, BT12 6BA UK; 5 Regional Virus Laboratory, Belfast Health and Social Care Trust, Belfast, Northern Ireland, BT12 6BA UK; 6 Institute for Global Food Security, School of Biological Sciences, Queen’s University Belfast, Belfast, Northern Ireland, BT9 5DL UK; 7 School of Pharmacy, Medical Biology Centre, Queen’s University Belfast, Belfast, Northern Ireland, BT9 7BL UK; 8 Current address: UCD National Virus Reference Laboratory, University College Dublin, Belfield, Dublin 4, D04 E1W1 Ireland

**Keywords:** COVID-19, SARS-CoV-2, Whole genome sequencing (WGS), Viral introductions, Phylogenomics, Genomic surveillance, Transmission dynamics, Viral phylogeography, Variant tracking, Public health

## Abstract

**Background:**

Ireland’s COVID-19 response combined extensive SARS-CoV-2 testing to estimate incidence, with whole genome sequencing (WGS) for genome surveillance. As an island with two political jurisdictions―Northern Ireland (NI) and Republic of Ireland (RoI)―and access to detailed passenger travel data, Ireland provides a unique setting to study virus introductions and evaluate public health measures. Using a substantial Irish genomic dataset alongside global data from GISAID, this study aimed to trace the introduction and spread of SARS-CoV-2 across the island.

**Methods:**

We recursively searched for 29,518 SARS-CoV-2 genome sequences collected in Ireland from March 2020 to June 2022 within the global SARS-CoV-2 phylogenetic tree and identified clusters based on shared last common non-Irish ancestors. A maximum parsimony approach was used to assign a likely country of origin to each cluster. The geographic locations and collection dates of the samples in each introduction cluster were used to map the spread of the virus across Ireland. Downsampling was used to model the impact of varying levels of sequencing and normalisation for population permitted comparison between jurisdictions.

**Results:**

Six periods spanning the early introductions and the emergence of Alpha, Delta, and Omicron variants were studied in detail. Among 4439 SARS-CoV-2 introductions to Ireland, 2535 originated in England, with additional cases largely from the rest of Great Britain, United States of America, and Northwestern Europe. Introduction clusters ranged in size from a single to thousands of cases. Introductions were concentrated in the densely populated Dublin and Belfast areas, with many clusters spreading islandwide. Genetic phylogeny was able to effectively trace localised transmission patterns. Introduction rates were similar in NI and RoI for most variants, except for Delta, which was more frequently introduced to NI.

**Conclusions:**

Tracking individual introduction events enables detailed modelling of virus spread patterns and clearer assessment of the effectiveness of control measures. Stricter travel restrictions in RoI likely reduced Delta introductions but not infection rates, which were similar across jurisdictions. Local and global sequencing levels influence the information available from phylogenomic analyses and we describe an approach to assess the ability of a chosen WGS level to detect virus introductions.

**Supplementary Information:**

The online version contains supplementary material available at 10.1186/s13073-024-01409-1.

## Background

The island of Ireland (Irish: Éire, referred to hereafter as Ireland) is located on the periphery of Northwestern Europe and offers a unique vantage point for studying the transmission dynamics and impact of infectious diseases. The island comprises two distinct political entities with a combined population of $\sim$7 million: Northern Ireland (NI) with a population of $\sim$1.9 million (Northern Ireland Statistics and Research Agency (NISRA; Irish: Gníomhaireacht Thuaisceart Éireann um Staitisticí agus Taighde) [1]), which is one of the nations of the United Kingdom (UK), and Ireland (referred to hereafter as Republic of Ireland (RoI)) with a population of $\sim$5.1 million inhabitants (Central Statistics Office (CSO; Irish: An Phríomh-Oifig Staidrimh) [2]). This geopolitical structure provides an opportunity to examine the effect of independent pandemic response measures on disease transmission across otherwise similar geographic regions.

In recent decades, Ireland has faced various public health challenges posed by emerging infectious diseases including outbreaks of influenza and the global H1N1 influenza A virus pandemic [3]. Understanding how regional responses and interactions influence the emergence and transmission of infectious diseases is crucial for effective public health interventions. Ireland’s connectivity, not only within Europe and North America, but with over 40 countries by high-volume and frequent direct flights underscores the need for robust surveillance and response strategies to mitigate the spread of pathogens. Genomic surveillance has revolutionised our ability to track and understand the evolution of pathogens. This approach is instrumental in identifying transmission patterns and monitoring the spread of infectious agents both within and across borders.

The emergence of severe acute respiratory syndrome coronavirus 2 (SARS-CoV-2) provided an unprecedented opportunity for developing and implementing genomic surveillance programmes in Ireland. SARS-CoV-2 is a non-segmented, single-stranded positive-sense ribonucleic acid (RNA) virus in the family *Coronaviridae*, which was first detected in Wuhan, Hubei Province, China, in December 2019, with an initial draft genome posted in early January 2020 [4, 5]. The virus is characterised by having an unusually large genome ($\sim$30 kbp) for an RNA virus but is typical of coronaviruses [6].

Despite the presence of an error-correcting RNA-dependent RNA replicase [7], which reduces the overall mutation rate (e.g. compared to human immunodeficiency virus or influenza viruses), SARS-CoV-2 still mutates with an estimated rate of about $1\times 10^{-6}$ to $2\times 10^{-6}$ mutations per nucleotide per replication cycle [8–10]. RNA editing carried out by host proteins such as members of the ADAR gene family deaminating adenosine (A) to inosine and apolipoprotein B mRNA-editing catalytic polypeptide-like (APOBEC) gene family deaminating cytosine (C) to uracil (U) result in pervasive A$\rightarrow$guanine (G) and C$\rightarrow$U mutations [11, 12]. This accumulation of viral mutations leads to an estimated substitution rate of about two novel single-nucleotide polymorphisms appearing each month [10, 13, 14]. The continuous evolution of SARS-CoV-2 results in the sporadic emergence of new viral lineages, which can evade naturally acquired immunity and diminish the effectiveness of existing vaccines [15]. New lineages are often characterised by many novel mutations that have accumulated rapidly prior to detection [16] or, more rarely, by RNA recombination events [17, 18].

Whole genome sequencing (WGS) for genomic surveillance facilitates the identification of viral variants, enabling viral transmission to be tracked in epidemics and pandemics [19–21]. Knowledge of circulating SARS-CoV-2 variants informs public health policies and responses [22]. In response to the pandemic, the COVID-19 Genomics UK (COG-UK) consortium was established in April 2020 to provide high-throughput WGS of SARS-CoV-2 RNA extracted from positive cases in the nations of the United Kingdom (UK) [23]. As COG-UK members, we were responsible for sequencing the majority of Northern Irish SARS-CoV-2 samples [24].

In RoI, SARS-CoV-2 WGS was predominantly performed by the National Virus Reference Laboratory in University College Dublin on behalf of the National SARS-CoV-2 Surveillance & WGS programme. The high per-capita SARS-CoV-2 WGS programmes within the island of Ireland provide an opportunity to study viral introductions into and spread between its two most populous urban centres (Belfast and Dublin) and the remainder of the island. Note that for the purposes of this study, the designation ‘Irish’ is used henceforth to describe all samples collected in Ireland.

The transmission and severity of SARS-CoV-2 have been shown to be influenced by various human factors, including population density and regional deprivation. Studies have identified population density as a potential factor in the transmission of the virus, with variations observed across different regions of NI [25]. Moreover, the risk of dying due to SARS-CoV-2 infection has been found to be associated with regional deprivation [26].

Deprivation is a primary risk factor for SARS-CoV-2 deaths among care home residents in England [27]. Furthermore, the mortality rate for deaths involving SARS-CoV-2 has also been shown to be significantly higher in the most deprived areas of England [28]. Additionally, socioeconomic deprivation was linked to a higher rate of critical cases and mortality due to SARS-CoV-2 in Scotland [29]. These findings suggest that regional deprivation is a key factor that must be considered when assessing the impact of the pandemic within Ireland.

In NI, there is already evidence linking SARS-CoV-2 incidence rate to deprivation data [25]. The identification of SARS-CoV-2 introductions and monitoring of viral spread allows us the opportunity to investigate their links with deprivation. Such an approach may help in identifying areas in Ireland that are at higher risk of transmission and mortality, and inform targeted public health interventions. By identifying areas that are most vulnerable to the virus, policymakers and healthcare professionals can develop targeted interventions and allocate resources more effectively, ultimately reducing pandemic harm.

Multifarious studies have characterised the initial introduction of SARS-CoV-2 to specific regions [30–54] or variants of SARS-CoV-2 to regions [55–67]. A previous phylogenetic analysis of 225 Irish genomes from two pandemic waves suggested that multiple introductions of SARS-CoV-2 to Ireland from outside had taken place [48]. However, the extent and originating locations of these and other introductions to Ireland remain to be elucidated. In this study, we aim to use the data now available to investigate the introductions to Ireland.

**Fig. 1:**
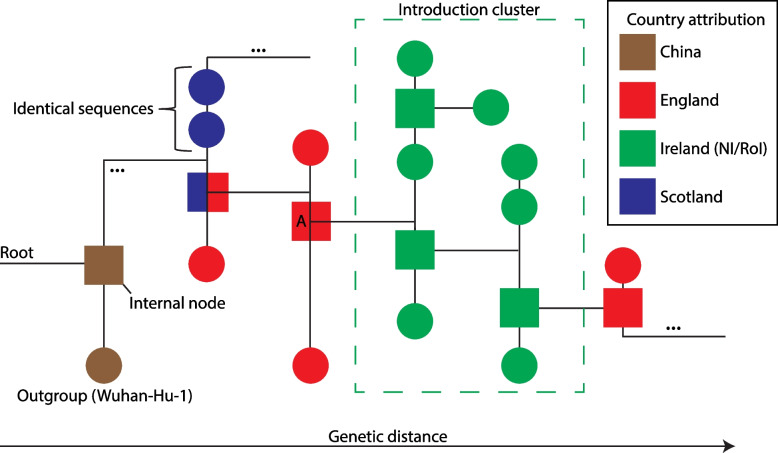
Schematic phylogeny. An abstract representation of a phylogeny illustrating an Irish introduction cluster as analysed in this study. The ancestral node to the introduction cluster is emphasised with a red square (indicating inferred origin from England) and is marked with an ‘A’. An internal node of ambiguous origin is depicted upstream of the ancestral node to the introduction cluster (‘A’), to which it could not be discerned whether the internal node descends from Scotland or England (see blue and red square)

Our first step in finding introduction events was to identify clusters of Irish samples within the global SARS-CoV-2 phylogeny (see Fig. 1). The country of origin attributed to the last common ancestor of an Irish cluster suggests the likely origin of the introduction. We leveraged a global SARS-CoV-2 phylogeny of more than seven million sequences to identify such introductory events. During six key time periods throughout the pandemic, we quantified introductions ascribed to the following major viral lineages as designated by the World Health Organization (WHO) and Pango nomenclature [68] as assigned by the pangolin tool [69]: early initial variants, Pango B.1.177, Alpha (Pango B.1.1.7), and Delta (Pango B.1.617.2/AY), as well as two major Omicron lineages (i.e. Pango B.1.1.529/BA.1 and Pango BA.2). The results yield insights into SARS-CoV-2 epidemiology within Ireland during each analysed time period, including SARS-CoV-2 importation frequencies and the subsequent geographic spread of infections. The workflow presented here is generalisable (i.e. it can be applied to any geographic region and virus of interest given sufficient sequencing data) and therefore can inform and improve global public health responses to both present and future viral and other pathogens.

## Methods

### Data

#### SARS-CoV-2 phylogenetic tree

A global SARS-CoV-2 phylogeny [70] dated 20 May 2022 was downloaded from Global Initiative on Sharing All Influenza Data (GISAID) EpiCoV^™^ [71, 72] on 24 May 2022. This phylogenetic tree is based on the masked alignment [73] of 7,603,547 high coverage (i.e. having < 1% undefined bases (Ns)) SARS-CoV-2 genome sequences (Additional file 1: Fig. S1).

#### Introductions SARS-CoV-2 sequence metadata

Metadata including the date and country of sample collection for all GISAID sequences was downloaded on 4 June 2022. We have employed the GISAID nomenclature to attribute samples to countries.

#### SARS-CoV-2 confirmed cases

The number of daily confirmed SARS-CoV-2 cases for NI and RoI were downloaded from the coronavirus (COVID-19) in the UK dashboard [74] and Ireland’s COVID-19 Data Hub [75] respectively.

#### Passengers arriving to Ireland by air

Our study analysed aviation data pertaining to passengers travelling to Ireland between March 2020 and June 2022 as sourced from NISRA [76] for NI and CSO [77] for RoI. While CSO data for RoI was directionally resolved, allowing raw use of arriving passenger numbers, monthly arrival passenger populations for NI were estimated by halving the published passenger flow values (i.e. $\text {monthly arrivals} = \frac{\text {monthly passengers}}{2}$).

#### Passengers arriving to Ireland by sea

To monitor the arrival of short sea ferry passengers travelling from Great Britain to the island of Ireland, we considered routes originating from Cairnryan (Scotland), Fishguard (Wales), Holyhead (Wales), Liverpool (England), or Pembroke (Wales) and terminating at Belfast (NI), Dublin (RoI), Larne (NI), or Rosslare (RoI). We also compiled data on international sea passenger routes to Ireland, which included routes departing from Bilbao (Spain), Cherbourg (France), and Rosscoff (France). These routes led to Cork, Dublin, and Rosslare in RoI. To obtain the sea passenger numbers for RoI, we used data from CSO. Specifically, we utilised the sum of the air and sea monthly directionally resolved passenger data [78] and then subtracted the corresponding figures for air travel [77]. For NI, sea passenger data, which derives from the forms available publicly (SPAS0107 and SPAS0201) [79], was obtained upon request from the UK Department for Transport Aviation and Maritime Analysis division. This data includes only passengers between Great Britain and NI, excluding Crown Dependencies like the Isle of Man, and encompasses both leisure passengers and Heavy Goods Vehicle (HGV) freight drivers.

#### Population density for government districts in Ireland

Population data within RoI and NI were obtained from CSO [2] and NISRA [1], respectively. To compute the population densities, the population within each government district was divided by the corresponding geographic area of the district, as sourced by Ordnance Survey of Northern Ireland (OSNI) for NI [80] and Tailte Éireann for RoI [81]. See Additional file 1: Tab. S1 for the values used in the computation of population density.

#### Deprivation for government districts in Ireland

The population data of subgroups in NI are primarily available at the level of Super Output Areas (SOAs). These areas were developed by NISRA with the aim of improving small-area statistics, and they were first produced for the 2011 census outputs [82]. Multiple deprivation measures in SOAs are presented as rankings, with SOAs in NI ranked from 1 (most deprived) to 890 (least deprived) [83]. Ranked-based data is deemed unsuitable for measurements; thus, an indirect calculation of the level of deprivation is necessary using alternative data such as income level.

SOA data can be disaggregated into grid cells using the dasymetric technique [84]. As such, this method can be applied to disaggregate deprivation data (e.g. income level) from the SOA level into grid cells, generating grid-based deprivation data across NI. These outcomes can be subsequently merged with administrative areas to estimate the level of deprivation within those regions.

In NI, the proportion of the population residing in households with equivalised income below 60% of the NI median [83] at the SOA level is utilised as a metric for measuring deprivation (%). For this analysis, WorldPop data for the entirety of NI in 2020 is also utilised. The dataset is obtainable at a granular level of grid cells (100 m cell at the Earth’s equator) for both the UK and Ireland, with the units representing the number of individuals per cell [85]. To determine the number of individuals inhabiting each SOA (2020), population grid cells are utilised. Subsequently, the % of individuals living in deprivation is converted into the ‘number of deprived individuals in SOA’, and a straightforward algebraic expression is employed to devise a weighted overlay technique for disaggregating the number of deprived individuals into grid cells:

$$\begin{aligned} \frac{\text {Population value in grids}}{\text {Total population within SOA}} \times \text {number of deprived individuals in SOA} \end{aligned}$$

The results, which represent the number of deprived individuals in grid cells, are superimposed onto administrative boundaries to estimate the number of deprived individuals within each area. Subsequently, these figures are transformed into a percentage of deprived individuals within administrative areas, utilising total population data in each SOA.

Deprivation data (deprivation score) for RoI is accessible at the administrative level of county councils [86]. It is crucial to note that, in this study, the calculation of deprivation in NI and RoI is based on distinct methodologies, and therefore, direct comparisons between the two cannot be made.

#### Country centroid positions

World country centroid positions were downloaded from gavinr’s GitHub repository ‘World Countries - Centroids’ [87]. In addition to these, centroid positions were added individually for England (latitude (Lat): 52.561928 °N, longitude (Lon): 1.464854 °W), Scotland (Lat: 56.816738 °N, Lon: 4.183963 °W), Wales (Lat: 52.33022 °N, Lon: 3.766409 °W), and NI (Lat: 54.607577 °N, Lon: 6.693145 °W) [88] as well as for Taiwan (Lat: 23.973861 °N, Lon: 120.982 °E) [89], Hong Kong (Lat: 22.3453 °N, Lon: 114.1372 °E) [90], Réunion (Lat: 21.114444 °S, Lon: 55.5325 °E) [91], Canary Islands (Lat: 28.47707 °N, Lon: 15.745798 °W) [92], Palestine (Lat: 31.4 °N, Lon: 35 °E) [93], Crimea (Lat: 45.25 °N, Lon: 34.6 °E) [94], and Kosovo (Lat: 42.55 °N, Lon: 20.85 °E) [95].

#### Irish SARS-CoV-2 genomes and metadata for substitution rate estimation

The entire GISAID FASTA and metadata databases were downloaded on 18 February 2023 to analyse population-wide SARS-CoV-2 genomics in Ireland. These data were then filtered to extract Irish samples (i.e. country=‘Ireland’ or country=‘NorthernIreland’) using augur filter v21.0.1 [96, 97].

### Identifying and removing tree outliers

To identify samples that potentially have incorrect collection dates in their associated metadata or deviate substantially from their expected position in the tree under a molecular clock, the GISAID phylogenetic tree and sample collection dates were input to Chronumental v0.0.50 [98, 99] to estimate a time-tree from the phylogeny. The difference between sample collection dates and the date predicted by Chronumental was calculated and a *z*-score (i.e. the statistical standard score) was calculated for each sample. Samples with a *z*-score greater than + 3 or less than − 3 were excluded from further analysis. These *z*-scores equate to roughly a ± 60 days difference in date. This step excluded 0.6% (46,278/7,603,547) of samples in the tree (Additional file 1: Fig. S2).

### Creating subtrees

GoTree v0.4.3 [100, 101] was used to prune the GISAID phylogenetic tree to subtrees used in subsequent analyses.

### Filtering Northern Irish sample metadata

Due to inconsistencies between country and finer-grain location metadata, some NI samples were suspected to be erroneously labelled as being from NI. Inclusion of these samples could overestimate the number of identified introductions. Samples with an NIRE prefix identifier (ID) (e.g. NIRE-000107) should all be collected and sequenced in NI and correctly attributed a country location of NI. Samples with NIRE prefix IDs comprise 84.3% of NI samples in the phylogenetic tree, while RAND IDs comprise 8.2%, NORT IDs 2.7%, and 15 other prefixes total 4.8% of NI samples. Therefore, identified NI introductions were filtered to consider introductions that consisted of NIRE IDs only. This excluded 5,427 samples.

### Reconstructing ancestral location of lineages

To infer ancestral locations of samples, a phylogenetic tree and the country of each sample in the tree was provided to PastML. Locations, at the level of countries, were inferred for each ancestral node of the phylogenetic tree (internal points between tips and the root of the tree that represent an ancestral state) using the DELTRAN (delayed transformation) method [102] in PastML v1.9.34 [103, 104]. This method prioritises minimising state ambiguities by implementing character changes as close to the tips of the phylogenetic tree as possible, thus favouring parallel mutations. Additionally, the –resolve_polytomies PastML option was enabled; this optional algorithm iteratively identifies nodes with more than two children (‘polytomies‘) and introduces new internal nodes to group children by their predicted ancestral location states, ensuring the tree structure more accurately reflects evolutionary relationships.

To assess the robustness of our selected ancestral state reconstruction method, we conducted supplementary analyses comparing the DELTRAN maximum parsimony method with TreeTime [105], a maximum likelihood method. Comparisons of these different ancestral state reconstruction approaches are presented (Additional file 1: Fig. S3), demonstrating consistency in introduction allocations among the methods tested. Detailed results are provided in within Additional file 1 (i.e. Tables S2–S7 may be cross-referenced with Tables S8–S13 for a comprehensive comparison).

### Clustering of sequences to identify independent introductions to Ireland

DendroPy v4.5.2 [106, 107] was used to traverse the output trees from PastML to identify Irish tips of the tree that were descended from ancestral nodes with a non-Irish country label assigned by PastML. We define all Irish tips linked to an importation event to comprise an introduction cluster.

The following periods were selected to span from prior to the first detected sequence of each major lineage in Ireland until they reached dominance.

#### Period A-Initial introductions

To identify initial introductions to Ireland, the tree was pruned to include only sequences from the start of the pandemic up to the end of May 2020 and rooted using Wuhan-Hu-1 (GISAID: EPI_ISL_402125) as an outgroup. Rooting a tree is the process of deciding which part of the tree is the oldest point and gives the direction of evolution moving from the root to the tips of the tree (Fig. 1). An outgroup, a tip or lineage outside the group of tips of interest yet related to the the group, allows the root to be determined. Here, we use one of the earliest SARS-CoV-2 sequences as the outgroup to the rest of sequences in the tree. This tree included 103,316 tips of which 714 are from RoI and 633 are from NI (Additional file 1: Fig. S4). Ancestral location of lineages was inferred by PastML as above. Location could not be resolved for 64 nodes (0.05%) and 2,053 new internal nodes were created by resolving polytomies.

#### Period B-B.1.177

To identify Pango [68] lineage B.1.177 introductions to Ireland, the tree was pruned to include only B.1.177.* sequences (and renamed sublineages of B.1.177.*: AA.1, AA.2, AA.3, AA.4, AA.5, AA.6, AA.7, AA.8, Z.1, Y.1, W.1, W.2, W.3, W.4, V.1, V.2, U.1, U.2, U.3) from the first non-outlier occurrence of B.1.177 to the end of October 2020 and rooted using Wuhan-Hu-1 (GISAID: EPI_ISL_402125) as an outgroup (Additional file 1: Fig. S5). This tree included 31,869 tips of which 215 are from RoI and 235 are from NI. Ancestral location of lineages was inferred by PastML as above. Location could not be resolved for 4 nodes (0.01%) and 364 new internal nodes were created by resolving polytomies.

#### Period C-B.1.1.7 - Alpha

To identify Pango lineage B.1.1.7 introductions to Ireland, the tree was pruned to include only B.1.1.7 sequences (and renamed sublineages of B.1.1.7.*: Q.1, Q.2, Q.3, Q.4, Q.5, Q.6, Q.7, Q.8) from the first non-outlier occurrence of B.1.1.7 to the end of February 2021 and rooted using Wuhan-Hu-1 (GISAID: EPI_ISL_402125) as an outgroup (Additional file 1: Fig. S6). This tree included 183,092 tips of which 2850 are from RoI and 461 are from NI. Ancestral location of lineages was inferred by PastML as above. Location could not be resolved for 45 nodes (0.02%) and 2181 new internal nodes were created by resolving polytomies.

#### Period D-B.1.617.2/AY - Delta

To identify Delta introductions to Ireland, the tree was pruned to include only B.1.617.2/AY sequences from the first non-outlier occurrence to the end of July 2021 and rooted using Wuhan-Hu-1 (GISAID: EPI_ISL_402125) as an outgroup (Additional file 1: Fig. S7). This tree included 512,420 tips of which 4923 are from RoI and 1613 are from NI. Ancestral location of lineages was inferred by PastML as above. Location could not be resolved for 287 nodes (0.05%), and 6363 new internal nodes were created by resolving polytomies.

#### Period E- B.1.1.529/BA.1 - Omicron

To identify Omicron introductions to Ireland, the tree was pruned to include only B.1.1.529/BA.1 sequences from the first non-outlier occurrence to the end of January 2022 and rooted using Wuhan-Hu-1 (GISAID: EPI_ISL_402125) as an outgroup (Additional file 1: Fig. S8). This tree included 1,313,634 tips of which 7888 are from the RoI and 3615 are from NI. Ancestral location of lineages was inferred by PastML as above. Location could not be resolved for 497 nodes (0.03%) and 22,546 new internal nodes were created by resolving polytomies.

#### Period F- BA.2 - Omicron

To identify BA.2 introductions to Ireland, the tree was pruned to include only B.2 sequences from the first non-outlier occurrence to the end of March 2022 and rooted using Wuhan-Hu-1 (GISAID: EPI_ISL_402125) as an outgroup (Additional file 1: Fig. S9). This tree included 644,494 tips of which 1235 are from RoI and 5136 are from NI. Ancestral location of lineages was inferred by PastML as above. Location could not be resolved for 338 nodes (0.05%) and 12,722 new internal nodes were created by resolving polytomies.

### Downsampling tips from England

To determine the effect of the relative over-representation of English samples in the tree on the high level of English introductions identified to Ireland, we randomly downsampled tips from England in the tree. Between 10 and 90% of English samples were randomly removed from the tree in 10% increments, with three replicates at each increment. Downstream analysis to identify introductions to Ireland was conducted as before.

### Downsampling tips from Ireland

To determine the effect of the level of sequencing in Ireland on the number and size of Irish introductions for the island as a whole and for each jurisdiction, we randomly downsampled tips from NI, RoI, and Ireland in the tree. Between 10 and 90% of samples from Ireland were randomly removed from the tree in 10% increments, with one replicate at each increment. Downstream analysis to identify introductions to Ireland and clusters size was conducted as before.

### Estimation of total vs. identified introductions

A goal of this study was to evaluate the number of genetic sequences that would be required to identify all SARS-CoV-2 introductions to Ireland. To determine the additional number of introductions ($I$) that could be identified by analysing more sequences ($S$), we utilised ordinary least-squares (OLS) linear regression function scipy.stats.linregress from the Python library SciPy [108].

For each period, let $\mathbb {S}$ be the total number of available sequences and $\mathbb {I}$ be the corresponding number of introductions to Ireland identified. During downsampling, it is appropriate to select $N$ (e.g. 10) evenly spaced downsampled sequencing values $s_i$ such that

$$\begin{aligned} s_i = i\bullet \frac{\mathbb {S}}{N} \quad \text {for}\ i=1,2,\ldots ,N, \end{aligned}$$

which yields an equivalent arraywhere $\Delta S \equiv \frac{\mathbb {S}}{N}$.

Through performing randomised downsampling of Irish tips in the phylogeny to each of the levels in array $S$ and detecting introductions for each, a corresponding array for introductions, $I$, will be obtained:where $I_1$ is the introductions predicted by downsampling to level $s_1$, etc.

A numerical difference array (with size of $N-1$) can be calculated from $I$:which enumerates the successively decreasing additional introductions identified upon increasing sequencing by $\Delta S$.

We suppose that these diminishing returns of increased sequencing will continue to hold, such that at a sequencing saturation point $\mathbb {S}_{\textrm{all}}$, all predicted introductions, $\mathbb {I}_{\textrm{all}}$, would be identified and there would be limited additional value to increasing sequencing levels.

To estimate $\mathbb {S}_{\textrm{all}}$, the sequencing level required to predict all introductions, we perform OLS linear regression to assess the relationship:

$$\begin{aligned}\Delta I = m \bullet S + c,\end{aligned}$$

where $m$ represents the slope of the linear regression line and $c$ represents the intercept of the linear regression line. Note that as $\Delta I$ has one element fewer than $S$, we must prepend an element to $\Delta I$, such that the first element contains the value $I_1$, as the number of additional introductions identified from zero sequencing to the level of $s_1$ is $I_1$.

To apply the regression equation for extrapolation, values for $S$ greater than the number of sequences obtained ($\mathbb {S}$) may be input to compute new values for $\Delta I$. Therefore, increased sequencing will provide additional introductions until $\Delta I = 0$, where $S=\mathbb {S}_{\textrm{all}}$, which is the intersection of the regression line with the $S$ axis: $\mathbb {S}_{\textrm{all}} = \frac{-c}{m}$ :

$$\begin{aligned} \Delta I = 0 = m \bullet \mathbb {S_{\textrm{all}}} + c \rightarrow \mathbb {S}_{\textrm{all}}=\frac{-c}{m}, \end{aligned}$$

thereby creating an upper bound for the extrapolation of $S$.

To calculate $\mathbb {I}_{\textrm{all}}$, extrapolated $S$ values ($S_{\textrm{extrapolation}}$) are firstly generated in an array from $\mathbb {S}+\Delta S$ to $\mathbb {S}_{\textrm{all}}$:

Inputting these ($S_{\textrm{extrapolation}}$) values into the regression equation, we similarly obtain $\Delta I_{\textrm{extrapolation}}$:

$$\begin{aligned}\Delta I_{\textrm{extrapolation}} = m \bullet S_{\textrm{extrapolation}} + c.\end{aligned}$$

Each extrapolated $\Delta I$ value within $\Delta I_{\textrm{extrapolation}}$ is summed with $\mathbb {I}$ to predict $\mathbb {I}_{\textrm{all}}$, the total number of introductions to Ireland within the period.

Note that our analysis assumes that the number of additional introductions is directly proportional to the total number of sequences. However, this assumption may not hold true, and future research in this area will need to be undertaken to determine the ideal functional form with which to perform such extrapolations.

### Geospatial mapping

GeoPandas v0.11.1 [109, 110] was used to facilitate geospatial visualisations of SARS-CoV-2 introductions to Ireland. As source data, two geospatial vector data files (i.e. shapefiles) were downloaded from the GADM Database of Global Administrative Areas [111], which aims to provide high-quality maps of the administrative areas of all countries, at all recognised levels of sub-division to be utilised freely for non-commercial use. The shapefile gadm41_IRL_1.shp as found within the IRL shapefile package [112] was used to map the counties of RoI, while gadm41_GBR_2.shp as found within the GBR shapefile package [113] was used to map the local government districts (LGDs (2014) [114]) of NI. The geographic resolution of RoI county and NI LGD is identical to the metadata fields accessible in GISAID or COG-UK metadata, allowing each sample to be associated to a distinct geographic region within Ireland. To create a map for the entire island of Ireland, NI was extracted from the shapefile containing the entirety of the UK and was merged with the shapefile containing RoI. To annotate the maps, Troendle–Rice–Simpson–Skvortsov 2-letter abbreviation codes were devised for each local government district as indicated in Additional file 1: Tab. S1. Samples with incomplete or ambiguous geographic metadata were excluded from geospatial mapping, resulting in 95% (31,529/33,131) of the Irish sequences being retained for these analyses.

### Spatial statistical analysis of Geographic Information System (GIS) data

Spatial statistical analyses of Geographic Information System (GIS) data were automated by the Spatial Statistics toolbox in the ArcGIS Pro v3.0.2 (Build 3.0.2.36056) software developed by Esri [115]. Geospatial data was input to the Spatial Statistics toolbox in ArcGIS Pro in the form of shapefiles, geodatabases, or feature classes, containing both the attribute and location data of the features being analysed.

The ordinary least squares (OLS) functionalities used within the ArcGIS Pro Spatial Statistics toolbox provide the results of bivariate regression of two independent variables: population density, and deprivation in correlation with the dependent variable: the proportion of Irish tips sequenced in the region linked to introduction and spreading events within the periods studied. The specific statistical measures revealed by the OLS functionalities are *p*-values, robust *p*-values, adjusted *R*^2^s, variance inflation factors (VIFs), and Koenker (BP) statistic (i.e. Koenker’s studentised Bruesch-Pagan statistic) probabilities. The number of regions used to analyse spatial statistics in RoI was 26 (one per county, with the four administrative areas in the Dublin region—Dublin City, Dún Laoghaire-Rathdown, Fingal, and South Dublin—merged into one, ‘County Dublin’), while for NI, there were 11 regions (one per LGD).

Before conducting bivariate regressions, we performed OLS regression to examine the relationship between population density and deprivation in NI and RoI. The results indicated minimal risk of overadjustment bias [116], as the correlation between population density and deprivation was weak (regression coefficient = 0.00085, *p*-value = 0.04 for RoI; regression coefficient = 0.002963, *p*-value = 0.0031 for NI) at the geospatial resolution studied. These correlations are visualised as bivariate sequential choropleth maps (9-class) (Additional file 1: Fig. S10).

### SARS-CoV-2 genomic trends in Ireland

We separately aligned each Irish genomic sequence from GISAID with Pango lineages corresponding to those of the six periods studied (*N *= 140,121) to the Wuhan-Hu-1 reference genome (GISAID: EPI_ISL_402125) using mafft v7.453 [73]. The number of substitutions in each Irish genome compared to the Wuhan-Hu-1 reference genome was determined by calculating the Hamming distance [117], which tallies the minimum number of substitutions required to change the reference sequence into a subject sequence of equal length, while excluding the contributions of insertions, deletions, and Ns in the subject sequence.

To analyse substitution rates per period, we conducted ordinary least squares (OLS) linear regression using the LinearRegression model from scikit-learn v1.1.3 [118, 119]. The data in Additional file 1: Tab. S14 includes error estimates and *p*-values from regressions that were nearly identical but conducted using the OLS functionality in statsmodels v0.13.5 [120, 121].

## Results

**Fig. 2:**
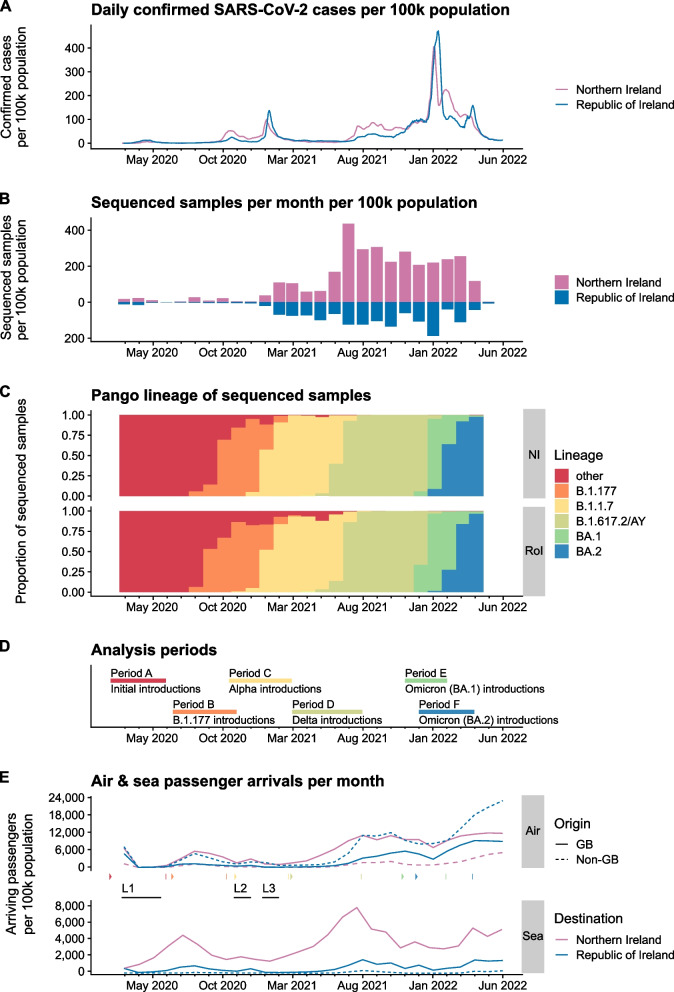
SARS-CoV-2 timeline in Northern Ireland and Republic of Ireland. **A** Seven day rolling daily mean of confirmed SARS-CoV-2 cases per 100,000 population. Data from coronavirus (COVID-19) in the UK dashboard and Ireland’s COVID-19 Data Hub. **B** Monthly histogram by collection date of SARS-CoV-2 genome sequences deposited to GISAID for Northern Ireland (NI) and Republic of Ireland (RoI). **C** Pango lineage of genome sequences deposited in GISAID for NI and RoI. Major lineages highlighted, all other lineages grouped under ‘other’. **D** Analysis periods used in this study to identify SARS-CoV-2 introductions to the island of Ireland. **E** Maritime and aviation passenger arrival volumes to NI and RoI per month. Lockdowns in Ireland are represented by three periods (L1-3) that occurred from late March to mid May 2020, late October to December 2020, and late December 2020 to February 2021, respectively. Although there were slight variations in the particular measures implemented between NI and RoI, these dates were selected to demonstrate a general agreement on lockdown periods between the two regions. The starts ($\rhd$) and the ends (|) of the periods studied are indicated

We began our study in Ireland by comparing SARS-CoV-2 case and sequencing rates, categorising lineages to determine key periods, and observing passenger volumes by air and sea (Fig. 2). Confirmed SARS-CoV-2 case rates followed similar trends in NI and RoI, with some differences including more cases in NI in autumn 2021 and February 2022 and more in RoI in January 2022 (Fig. 2A), which may reflect disparities in viral epidemiology and/or pandemic response measures between the two jurisdictions. The number of samples sequenced per month was mirrored between NI and RoI, with the per capita rate being higher in NI (Fig. 2B). Successive waves of dominant SARS-CoV-2 Pango [68] lineages were very similar, with a few other lineages persisting in RoI after the introduction of Alpha and a delayed rise in Omicron BA.2 in NI (Fig. 2C). The introduction of these lineages to the island of Ireland during each of the denoted periods (Fig. 2D) were analysed. Arriving air and sea passenger volumes (Fig. 2E) in both RoI and NI drastically reduced during the three major lockdowns (L1–L3) but began recovering to pre-pandemic levels from mid-2021.

### SARS-CoV-2 introductions from Europe and United States of America (USA) seeded early pandemic

Despite the relatively low numbers of sequenced samples in both RoI and NI from 2020 (Fig. 2B), some insights can be gleaned about the nature of these early importations. The first analysis period (Period A) is the beginning of the pandemic and aims to quantify the initial introductions of SARS-CoV-2 to the island of Ireland (Fig. 2C, D). The prepared global SARS-CoV-2 phylogenetic tree (see *SARS-CoV-2 phylogenetic tree*, *Identifying and removing tree outliers*, and *Filtering Northern Irish sample metadata*) was pruned to include only sequences from the start of the pandemic up to the end of May 2020 (see *Creating subtrees*). This tree included 103,316 tips of which 714 are from RoI and 633 are from NI. A country was estimated for each ancestral lineage of the phylogenetic tree using the DELTRAN (delayed transformation) method [102] (see *Reconstructing ancestral location of lineages*). SARS-CoV-2 introductions to Ireland could then be inferred by Irish sequences that are descendants of a non-Irish ancestral node in the tree (see *Clustering of sequences to identify independent introductions to Ireland*).

**Fig. 3:**
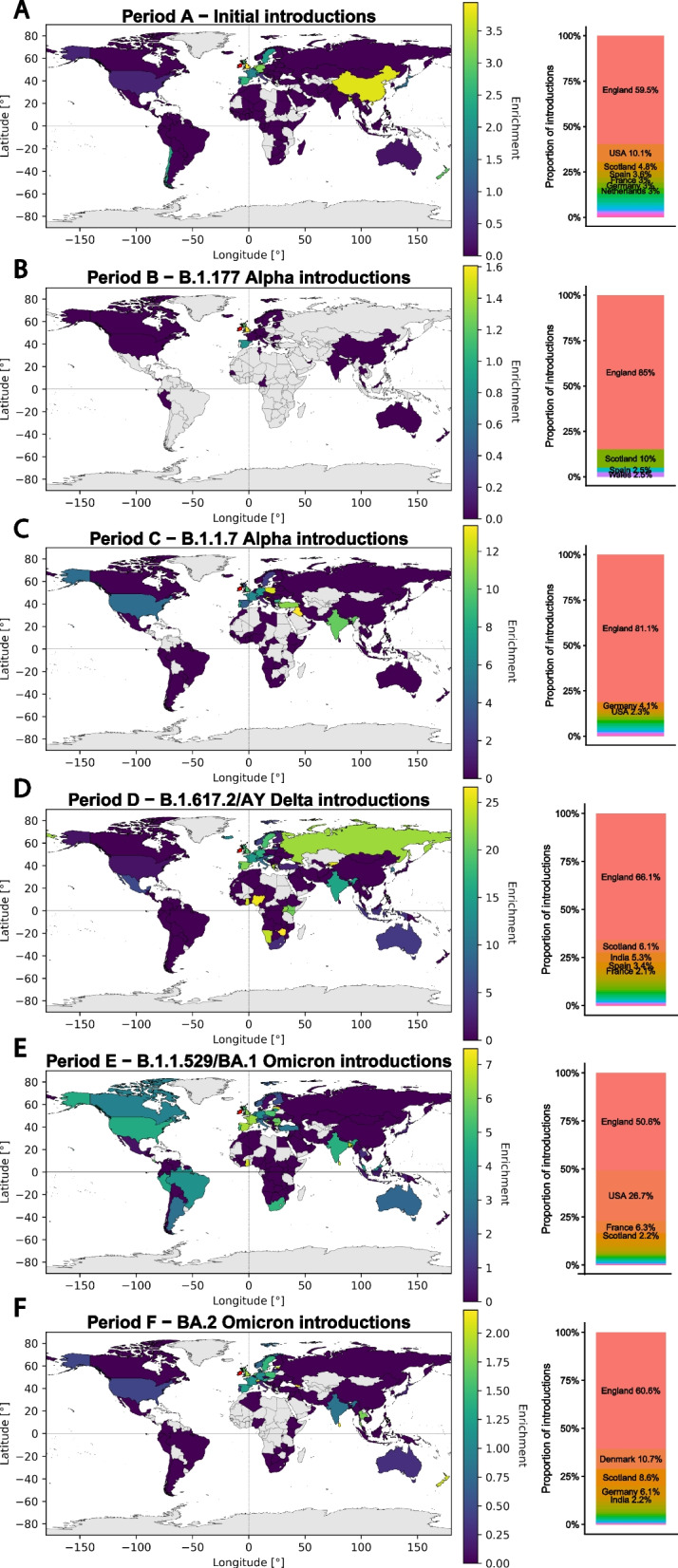
SARS-CoV-2 introductions. Left: for each period (**A**–**F**), a world map depicting the ratio of introductions to Ireland given the proportion of samples in the global phylogeny. World maps were generated with the public domain Natural Earth map dataset [122] using the GeoPandas v0.11.1 library [109, 110]. Note that the colour bar scales differ across the time periods examined. Furthermore, countries displayed in grey indicate absence in the GISAID-derived global phylogeny. Relative sequencing enrichment is defined: $\text {Enrichment} = \frac{\text {proportion of introductions to Ireland}}{\text {proportion of tips in the global phylogeny}}$ Right: for each period (**A**–**F**), the proportion of introductions originating from each country

We estimated that at least 170 independent introductions of SARS-CoV-2 took place in the time period up to the end of May 2020, 103 to RoI and 67 to NI, with three intra-Irish introductions, two from RoI to NI and one from NI to RoI. The majority of introductions originated from Europe, with 59.5% of introductions from England, 10.1% from United States of America (USA), and 4.8% from Scotland (Fig. 3 Period A right, Additional file 1: Tab. S2). There was a significant positive correlation between the number of introductions from a country and the number of tips in the tree from a country ($\rho = 0.75$, $P = 0.002$, Spearman’s rank correlation; Additional file 1: Fig. S11 Period A centre). There was no correlation between the number of introductions from a country and its distance from Ireland ($\rho = -0.47$, $P = 0.45$, Spearman’s rank correlation; Additional file 1: Fig. S11 Period A right).

### B.1.177 and Alpha introductions originated predominantly from England

The second analysis period (Period B) focuses on the emergence of Pango lineage B.1.177 over summer 2020 until the end of October 2020 by which time B.1.177 had become dominant in Ireland (Fig. 2C, D). The phylogenetic tree was pruned to contain B.1.177 sequences from this time and included 31,869 tips, of which 215 were from RoI and 235 were from NI. There were 41 introductions identified, of which 23 were into NI and 18 into RoI (Additional file 1: Tab. S3). Almost all introductions originated from Great Britain, with 85% of introductions from England (Fig. 3 Period B right). With only four unambiguous countries (England, Scotland, Wales, and Spain) for all importations in this period, no meaningful correlation with number of tips in the phylogenetic tree and distance from Ireland could be performed (Additional file 1: Fig. S11 Period B centre and right).

Period C focuses on introductions of B.1.1.7 Alpha lineage until the end of February 2021 (Fig. 2C, D). The phylogenetic tree was pruned to 183,092 tips of which 2,850 were from RoI and 461 were from NI. There were 173 introductions into RoI from outside Ireland, and 45 into NI (Additional file 1: Tab. S4). Additionally, there were 25 introductions between RoI and NI (14 from NI and 11 from RoI). The majority of introductions originated from England (81.1%), followed by Germany (4.1%), and USA (2.3%; Fig. 3 Period C right). While there was no correlation between the number of introductions and the distance from Ireland, there was a significant positive correlation between the number of introductions from a country and the number of tips in the tree from a country ($\rho = 0.85$, $P = 0.0003$, Spearman’s rank correlation; Additional file 1: Fig. S11 Period C right and centre, respectively).

### Delta lineage imported from England, Europe and India

Period D focuses on introductions of Delta lineages (Pango lineages B.1.617.2/AY) until the end of July 2021 (Fig. 2C, D).

The phylogenetic tree was pruned to 512,420 tips of which 4923 are from RoI and 1613 are from NI. There were 454 introductions into RoI from outside Ireland, 273 into NI, and one introduction to either RoI/NI (Additional file 1: Tab. S5). Additionally, there were 73 introductions between RoI and NI (31 from NI and 42 from RoI). The majority of introductions originated from England (66.1%), followed by Scotland (6.1%), India (5.3%), and Spain (3.4%; Fig. 3 Period D right). Interestingly, these are not balanced between NI and RoI. There were 228 introductions from England to NI (83.5% of NI introductions) yet only 224 introductions to RoI (49.3% of RoI introductions). Conversely, there were 33 introductions from India to RoI (7.2% of RoI introductions), and 23 from Spain (5.1%) yet only 5 introductions from India to NI (1.8% of NI introductions) and 1 from Spain (0.4%).

For Period D, we found a significant positive correlation between the number of introductions from a country and the number of tips in the tree from that country ($\rho = 0.77$, $P = 1.4 \times 10^{-6}$, Spearman’s rank correlation (Additional file 1: Fig. S12 Period D centre). There was also a significant correlation between the number of introductions from a country and the distance of each country from Ireland ($\rho = -0.65$, $P = 0.0004$, Spearman’s rank correlation; Additional file 1: Fig. S12 Period D right).

### England and USA account for majority of BA.1 introductions

Period E focuses on introductions of the Omicron (BA.1) lineage (Fig. 2C, D).

The phylogenetic tree was pruned to include 1,313,634 tips of which 7888 were from RoI and 3615 were from NI. There were 1937 introductions to Ireland (1329 introductions into RoI and 608 into NI) and 69 introductions between RoI and NI (31 from NI and 38 from RoI; Additional file 1: Tab. S6). About half of introductions were from England (50.8%), followed by USA (26.7%), and France (6.3%; Fig. 3 Period E right).

We found a significant positive correlation between the number of introductions from a country and the number of tips in the tree from that country for Period E ($\rho = 0.79$, $P = 5.3 \times 10^{-10}$, Spearman’s rank correlation; Additional file 1: Fig. S12 Period E centre). There was also a significant correlation between the number of introductions from a country and the distance of each country from Ireland ($\rho = -0.51$, $P = 0.003$, Spearman’s rank correlation; Additional file 1: Fig. S12 Period E right).

### BA.2 introductions predominantly from Northern Europe

Period F focuses on introductions of the Omicron (BA.2) lineage (Fig. 2C, D).

The phylogenetic tree was pruned to include 644,494 tips of which 1235 were from RoI and 5136 were from NI. There were 1309 introductions to Ireland (428 introductions into RoI and 881 into NI) and 34 introductions between RoI and NI (29 from NI and 5 from RoI; Additional file 1: Tab. S7). England (60.6%), Denmark (10.7%), Scotland (8.6%), and Germany (6.1%) were the most frequently observed origin countries for importations (Fig. 3 Period F right).

For Period F, we found a significant positive correlation between the number of introductions from a country and the number of tips in the tree from that country ($\rho = 0.91$, $P = 6.8 \times 10^{-11}$, Spearman’s rank correlation; Additional file 1: Fig. S12 Period F centre). There was also a significant correlation between the number of introductions from a country and the distance of each country from Ireland ($\rho = -0.60$, $P = 0.006$, Spearman’s rank correlation; Additional file 1: Fig. S12 Period F right).

### Clusters comprising multiple Irish sequences vary greatly in size

**Fig. 4:**
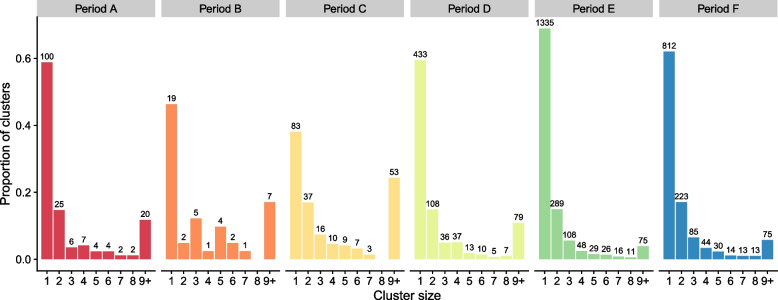
Irish samples per introduction. Number of Irish samples per introduction grouped by cluster size 1–9+ for periods A–F. Bar height shows the proportion of total clusters for each cluster size within a period

A cluster of multiple Irish sequences that arise from a single introduction event can be informative about virus spreading and epidemiology when combined with geographic or other information. However, 63.2% (2782/4403) of introductions across all six periods resulted in detection of only a single Irish descendant (Fig. 4), with the percentage at specific periods varying from 38.1% (Period C) to 68.9% (Period E) between periods studied. The largest cluster in each period ranged from 124 sequences in Period B to 2228 sequences in Period F. The large number of clusters having few samples implies that despite the globally high per capita sequencing rate in Ireland, the level of WGS sequencing may be insufficient to detect most cases spreading from each introduction and the number of importations will have been underestimated.

### Introductions from England exceed predictions based on sample numbers in the global phylogeny

Countries with an open, high-throughput variant surveillance programme will feature more frequently in the phylogenetic tree than other countries that have made few sequences publicly available. There is a strong relationship between the number of tips in the tree from a specific country and the number of introductions from the same country (Fig. 3). England is the most frequent origin country of importations to Ireland for all periods analysed. However, these high levels of importations observed could be due to England being a global leader in the SARS-CoV-2 sequencing effort by making many genome sequences available, rather than due to factors like the country being geographically close to Ireland with high connectivity.

**Fig. 5:**
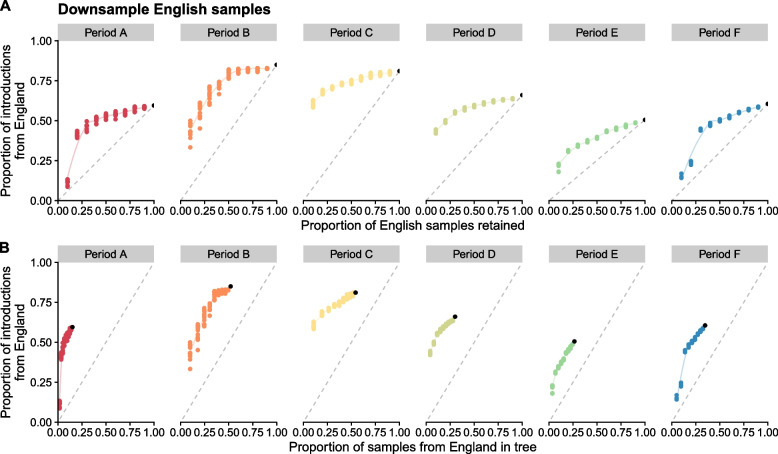
Effect of downsampling English samples. Ten replicates for each downsampled proportion. The black point is the number of observed introductions for each period using all data. Dashed grey lines represents a linear relationship between zero and observed introductions. The smoothed lines are fit to the downsampled data points using local polynomial regression fitting (loess). **A** Proportion of introductions to Ireland observed from England when English samples are randomly downsampled by 10% to 90%. **B** Proportion of introductions to Ireland observed from England when English samples are randomly downsampled and the proportion of samples in the phylogenetic tree that are from England. Three replicates for each downsampled proportion. Dashed grey line represents a linear relationship between 0% and 100%

To investigate the effect of sampling bias on the number of Irish importations attributed to England, we randomly downsampled tips from England in the tree to levels retaining between 90 and 10% of the original number (see *Downsampling tips from England*) and then counted importations to Ireland as before. If the proportion of importations from England to Ireland was due solely to the amount of English samples in the tree, we could expect a linear relationship when downsampling, i.e. reducing the number of English samples in the tree by 50% would reduce the proportion of introductions attributed to England by 50%. Instead, as samples are removed, we observe that the proportion of introductions from England remains higher than expected (Fig. 5A). This is significant for all six periods when compared to the linear expectation (*P* between $8.9 \times 10^{-8}$ and $3.6 \times 10^{-5}$ for each period; paired Wilcoxon signed-rank test). Additionally, the proportion of introductions from England to Ireland is always greater than the proportion of English samples in the tree for all downsampled replicates (Fig. 5B).

### Optimal Irish sequencing rates vary between periods

Different levels of sequencing impact the ability to detect introduction events throughout the pandemic. Increased SARS-CoV-2 sequencing in both RoI and NI in 2021 (Fig. 2B) yielded more rapid and informed surveillance of variants.

Sequencing at a very low level will miss whole clusters resulting from importations, and so when sequencing is low, the number of importations will be underestimated. At the other end of the spectrum, sequencing every SARS-CoV-2 case will detect all members belonging to each cluster, resulting in the best possible estimate of the number of importations. However, as the level of sequencing increases, the returns are diminishing because different members of the same cluster would be sampled, in which case the number of importations remains much the same.

**Fig. 6:**
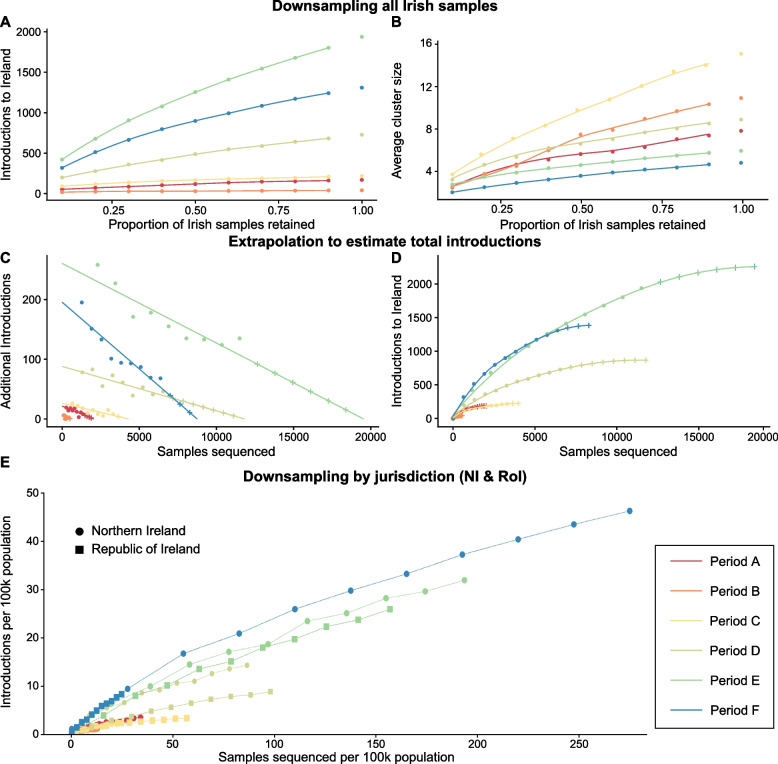
Effect of downsampling Irish samples (**A** and **B**), corresponding extrapolation to estimate total introductions to Ireland (**C** and **D**), and population normalised introduction and sequencing rates for NI and RoI (**E**). **A** Number of introductions to Ireland observed when Irish samples are randomly downsampled by 10% to 90%. One replicate for each downsampled proportion. **B** Average cluster size of Irish introductions when Irish samples are randomly downsampled by 10% to 90%. **C** The numerical derivatives of the downsampled data (**A**) were assessed and fit to a straight line to approximate the rate additional sequencing will have on finding additional introduction clusters. Estimated additional introductions at regular intervals are indicated by the symbol ‘+’. **D** The data is extrapolated from **C** revealing predicted sequencing saturation levels, where additional sequencing is unlikely to detect novel introduction events. Data using extrapolation are indicated by the symbol ‘+’. **E** Comparison of the number of introductions to NI and RoI for each period studied. Downsampling was performed independently for samples from each jurisdiction. To account for the difference in total populations, both the sets of downsampled sequences and identified introductions were normalised per 100k population

To determine the effect of different levels of sequencing on the number of Irish importations detected, we randomly downsampled Irish sequences in the tree, similar to above, retaining between 90 and 10% and then counted importations to Ireland as before (see *Downsampling tips from Ireland*). As performing additional replicates (*N *= 10) in Fig. 5 demonstrated little variation in the results, we determined that downsampling the number of Irish samples once per level for each period would be sufficient to capture the overall trends without substantially compromising the accuracy of our findings. For Periods A–C, removing 90% of Irish samples reduces the number of introductions detected to between 31 and 44% of the introductions observed (Fig. 6A). This is greater than the 10% expectation if introductions decreased linearly. For Periods D–F, a more steep decline is observed as samples are removed, and when only 10% remain, we detect between 22 and 27% of the original observed number of introductions. When average cluster size is considered, a steeper decline is instead observed for Periods A–C compared to D–F (Fig. 6B). Therefore, we hypothesise that if sampling was increased in Ireland beyond the level conducted for Periods A–C, few additional introductions would be detected, but instead most new samples would be added to existing introduction clusters and increase cluster size. For Periods D–F, an increasing in sampling would continue to detect many new introductions and increase cluster size of existing introductions.

It is a challenge for public health officials to assess the effectiveness of a given level of sequencing. To assist in this evaluation, we have developed a methodological framework involving the extrapolation of downsampled data to determine the proportion of total predicted introductions that were detected using the available sequencing (see *Estimation of total vs. identified introductions*). In this context, an ‘optimal’ sequencing level refers to the minimally required proportion to capture all estimated introduction events. The extrapolation results (see Table 1 and Fig. 6C and D) reveal these estimated number of sequences required to identify all introductions to Ireland. For Periods A–F, the number of introductions detected vs. predicted via extrapolation for these periods were 170/186, 41/42, 218/224, 728/867, 1937/2258, and 1309/1383, respectively. Similarly, the proportions of sequences available to the number estimated to detect all introductions were 1347/2044, 450/570, 3311/4303, 6536/11815, 11503/19538, and 6371/8751, respectively. Such extrapolations could provide a reliable approach for predicting the total number of introductions and required sequencing levels in comparable epidemiological scenarios (i.e. subject virus or geographic location).

**Table 1:** SARS-CoV-2 introductions detected vs. predicted and sequences identified vs. estimated sequences required for Periods A–F in Ireland

Period	Introductions detected vs. predicted		Sequences identified vs. estimated sequences required	
	Detected	Predicted	Identified	Required
A	170	186	1347	2044
B	41	42	450	570
C	218	224	3311	4303
D	728	867	6536	11815
E	1937	2258	11503	19538
F	1309	1383	6371	8751

In contrast to Fig. 6C, D which refer to the whole island, Fig. 6E shows the number of introductions to NI and RoI during Periods A–F. Normalising both introductions and sequences acquired per capita accounts for biases in total population and disparities in sequencing efforts. This reveals that introduction rates per capita were similar between NI (circles) and RoI (squares) with the exception of Period D during which they were higher in NI. This approach also allows comparison of sequencing levels per capita achieved during each period (rightmost points).

### Population density and deprivation significantly impact SARS-CoV-2 introductions and spread in Ireland

We examined the effect of population density and deprivation on the spread of the virus within NI and RoI using six ordinary least-squares (OLS) regression models and Geographic Information Systems (GIS) (see *Spatial statistical analysis of Geographic Information System (GIS) data*). Considering all regression models (see Table 2), variance inflation factors (VIFs) were below 3, indicating that there is no significant multicollinearity among the independent variables. Furthermore, the Koenker (BP) statistics are all above the 0.05 threshold, which suggests that the models do not suffer from heteroskedasticity, and indicate that applying geographically weighted regression (GWR) would not yield much additional clarity. Analysis of Periods D–F, which had the highest sequencing rates, revealed that both population density and deprivation had some statistically significant correlations with the introduction and spread of the virus.

**Table 2:** Summary of OLS regression and GIS results for population density and deprivation in Ireland

Period and country	Variable	Coefficient	Robust *p*	Adjusted $R^2$	VIF	Koenker (BP) statistic
D NI	Pop. density	2.4 × 10^−5^	6.7 × 10^−2^	8.7 × 10^−1^	2.8	0.38
	Deprivation	1.5 × 10^−2^	*2.4 × 10^−3^		2.8	1.7
E NI	Pop. density	− 6.0 × 10^−6^	7.3 × 10^−1^	5.2 × 10^−1^	2.8	0.09
	Deprivation	1.2 × 10^−2^	*2.5 × 10^−2^		2.8	1.5
F NI	Pop. density	− 2.0 × 10^−6^	9.4 × 10^−1^	2.7 × 10^−1^	2.8	0.13
	Deprivation	8.6 × 10^−3^	2.0 × 10^−1^		2.8	0.80
D RoI	Pop. density	2.6 × 10^−4^	*$\sim$0	9.6 × 10^−1^	1.2	0.48
	Deprivation	− 2.9 × 10^−3^	6.5 × 10^−1^		1.2	1.1
E RoI	Pop. density	8.3 × 10^−5^	*$\sim$0	3.1 × 10^−1^	1.2	0.84
	Deprivation	− 2.9 × 10^−3^	8.0 × 10^−1^		1.2	0.88
F RoI	Pop. density	1.7 × 10^−4^	*$\sim$0	8.9 × 10^−1^	1.2	0.18
	Deprivation	− 1.5 × 10^−2^	*2.1 × 10^−2^		1.2	1.32

**Fig. 7:**
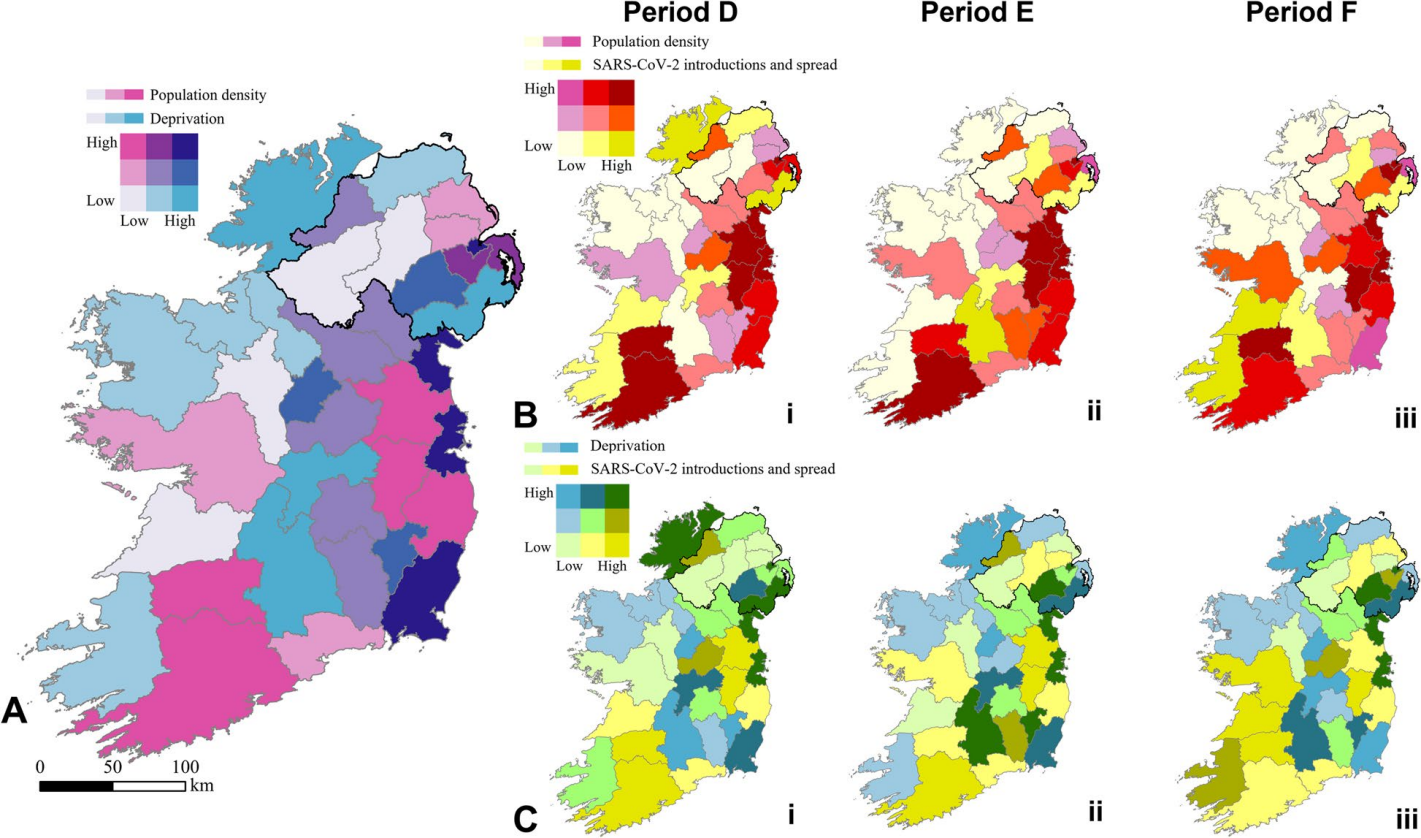
Bivariate sequential choropleth maps (9-class) depicting relationships between population density, deprivation, and SARS-CoV-2 introductions and spread for all local government districts in Ireland. **A** The association between population density (cyan palette) and deprivation (magenta palette) in Ireland. It is assumed that this relationship remains constant throughout the periods studied. **B** The correlations between population density (cyan palette) and the proportion of samples linked to the introduction and spread of SARS-CoV-2 (dark yellow palette). The three maps labelled i–iii correspond to periods D–F, respectively. **C** The correlations between deprivation (magenta palette) and the proportion of samples linked to the introduction and spread of SARS-CoV-2 (dark yellow palette). The three maps labelled i–iii correspond to periods D–F, respectively

Our GIS analysis indicated that the introduction and spread of SARS-CoV-2 infections was not randomly distributed across Ireland (Fig. 7). Population density displayed statistically significant positive correlations with SARS-CoV-2 introductions and spread in Period D (*p* < 0.05) in NI and in RoI (*p* < 10^−4^) during all three periods (D–F). More SARS-CoV-2 sequences were produced in more deprived areas of NI in Periods D and E (*p* < 0.05). However, a significant inverse relationship was observed with deprivation during Period F (*p* < 0.05) in RoI. This unexpected result may reflect differing sequencing strategies between NI and RoI. Also, note that these findings are correlational in nature and do not necessarily indicate causation.

You may interactively explore our datasets associating population density and deprivation in Ireland to SARS-CoV-2 introductions and spread [123].

### The localised SARS-CoV-2 spreading revealed by geospatiotemporal analysis of introduction clusters is consistent with our phylogenomic analysis

**Fig. 8:**
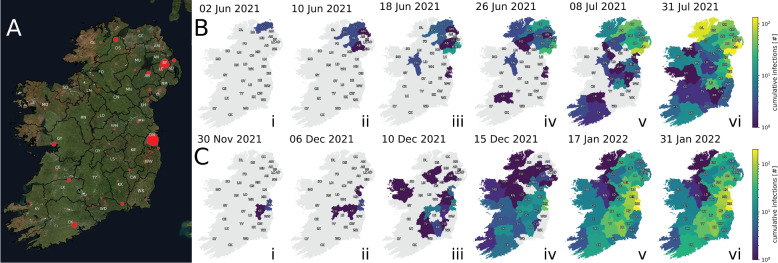
Geospatial tracking of the spread of samples from introduction events within Ireland. **A** A map of Ireland created with GeoPandas v0.11.1 ([109, 110]) using the Esri ’World Imagery’ basemap (Sources: Esri, DigitalGlobe, GeoEye, i-cubed, USDA FSA, USGS, AEX, Getmapping, Aerogrid, IGN, IGP, swisstopo, and the GIS User Community) as retrieved using contextily v1.2.0 [124]. Red points designate the locations of population centres in each government district with the size of each point scaled according to recent population estimates. Refer to Additional file 1: Tab. S1 for the corresponding metadata for region abbreviations and population centres. **B**-i Delta SARS-CoV-2 from Scotland begins a cluster in NI (Causeway Coast and Glens) on June 2, 2021. **B**-ii The cluster spreads to adjacent districts in NI by June 10, 2021. **B**-iii The cluster spreads to County Roscommon and County Dublin in RoI and adjacent districts in NI by June 18, 2021. **B**-iv The cluster reaches the rest of NI, County Donegal in RoI, and County Limerick in RoI by June 26, 2021. **B**-v Spread continues within NI and to additional regions in RoI by July 8, 2021. **B**-vi Spread across Ireland continues, affecting at least 960 individuals by July 31, 2021. **C**-i BA.1 SARS-CoV-2 from England is detected in County Dublin and County Kildare in RoI on November 30, 2022. **C**-ii The cluster spreads to County Offaly and County Louth by January 6, 2022. **C**-iii The cluster rapidly spreads to adjacent districts in RoI, two districts in NI, and County Mayo in RoI by December 10, 2021. **C**-iv The cluster continues spreading, reaching several districts in NI and RoI by December 15, 2021. **C**-v Infections linked to this cluster reach all districts in Ireland by January 17, 2022. **C**-vi Spread across Ireland continues until the last sequenced case on January 31, 2022, afflicting at least 1274 individuals over 62 days

We conducted geospatiotemporal analyses of introduction clusters by tracking the geographic appearance of phylogenetically descendent samples linked to each introduction event over time. Figure 8A depicts Ireland with local government districts of NI and RoI, while Fig. 8B and C illustrate the spreading patterns of the third and second-largest detected introduction clusters, respectively. An introduction cluster of 960 Irish Delta SARS-CoV-2 sequences originating from Scotland via Causeway Coast and Glens in NI during Period D (2 June–31 July 2021) showed intra- and neighbouring-region spread, with a transmission hop from NI to County Roscommon and County Dublin (Fig. 8Bii to iii). An even larger introduction cluster of 1274 Irish Omicron BA.1 SARS-CoV-2 sequences during Period E (30 November 2021–31 January 2022) spread similarly, originating from England via County Dublin and County Kildare (Fig. 8C (i–vi)).

The examples in Fig. 8 demonstrate how, when plotted temporally, the locations of cases within a cluster follow a non-random distribution, consistent with the expected scenario of predominantly local transmission events. Because the phylogenetic analysis of introduction clusters was performed with no reference to local spatial information (below country level), the existence of this relationship supports the veracity of the phylogenetically determined clusters.

Additional regional metadata, including 2-letter abbreviations for local government districts in Fig. 8 and information regarding population centres in each district, is provided in Additional file 1: Tab. S1. Additional file 1 includes longitudinal depictions of exemplary clusters involving fewer sequences (Additional file 1: Fig. S13) and references animations depicting the introduction and spread of these and the three largest clusters detected, including the largest detected cluster of BA.2 SARS-CoV-2 within Period F, which affected 2377 individuals and lasted 83 days in Ireland (see Additional file 1: Fig. S19 [125]).

### Nucleotide substitution rates in Ireland are consistent with global trends

We assessed SARS-CoV-2 substitution rates over time among the variants. This enabled comparison with other reports to identify any effect of Ireland’s unique geographic and cultural position and confirmation that there were no differences between NI and RoI which might affect detection of introductions. Our analysis demonstrated that the substitution rates of Irish SARS-CoV-2 were consistent with those earlier reported [9, 10] and were similar to the substitution rates estimated by Nextstrain [96, 126].

**Fig. 9:**
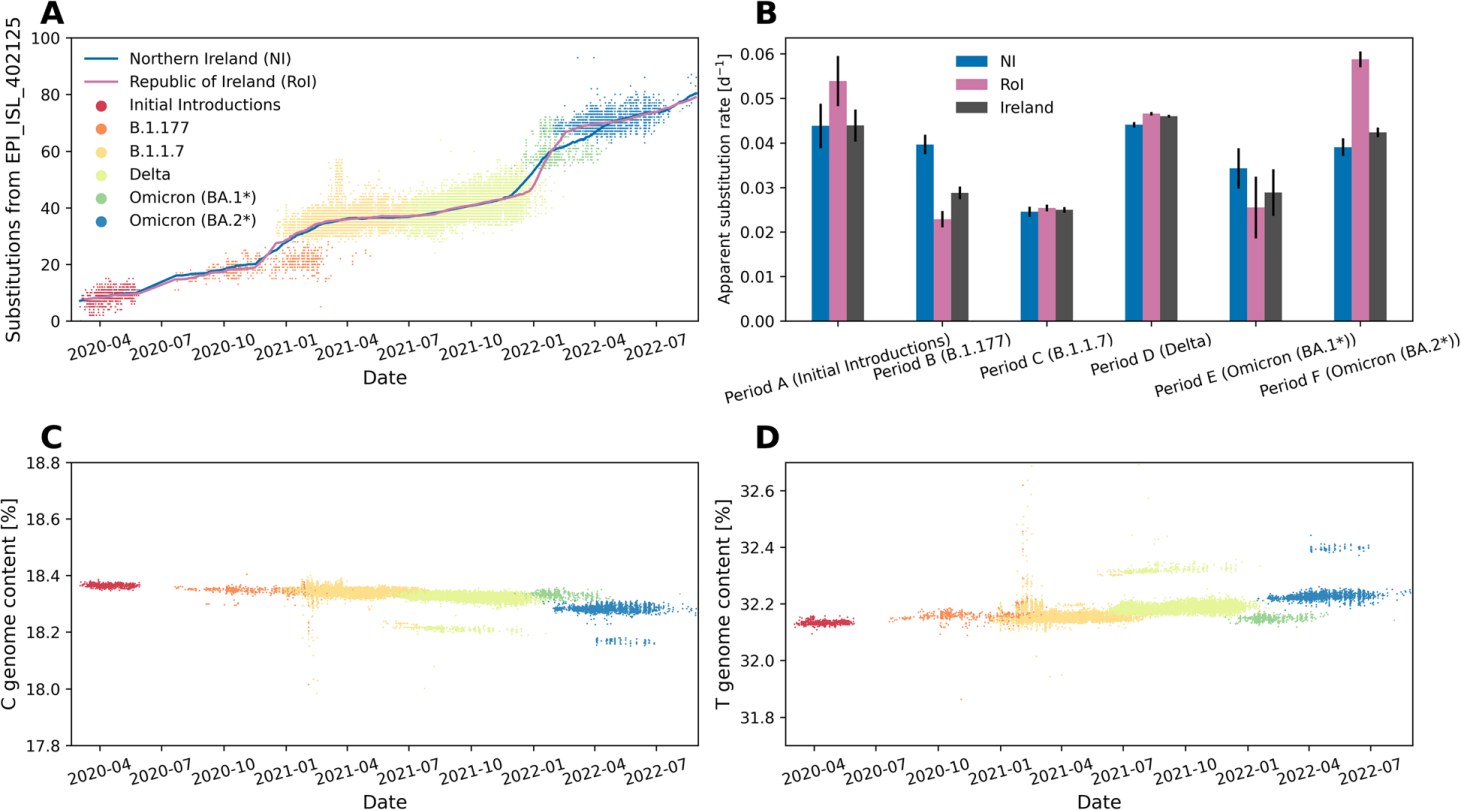
Population-wide SARS-CoV-2 genomic trends in Ireland. Genome coverage for all samples included were at least 99.9% upon alignment to the Wuhan-Hu-1 SARS-CoV-2 reference (GISAID: EPI_ISL_402125). **A** Observed substitutions from the Wuhan-Hu-1 SARS-CoV-2 reference per sequence. **B** Apparent substitution rates per major SARS-CoV-2 lineage related to each studied introduction period. See Additional file 1: Fig. S14 for a visualisation of each linear regression, and Additional file 1: Tab. S14 for the corresponding statistics. **C** Genome C content per sequence. **D** Genome T content per sequence

Figure 9A shows all (as of 18 February 2023) Irish sequences with $\ge$ 99.9% genome coverage as obtained from GISAID corresponding to lineages introduced in Periods A–F, with the mean observed substitutions tracked for NI and RoI as solid lines. We observe increases at transition periods due to the appearance of new lineages alongside the existing circulating variant (see Markov et al. [10] for a detailed explanation of this phenomenon). To investigate substitution characteristics of each lineage, we separately analysed the apparent substitution rates corresponding to lineages introduced in each period (see Additional file 1: Fig. S14). Estimated substitution rates for NI and RoI for each lineage group are presented in Fig. 9B. From this analysis, we can see that overall there do not appear to be significant differences between the apparent substitution rates of SARS-CoV-2 in NI compared to RoI. However, Period B, which is a time period with the lowest sequencing rates both locally and globally (see Fig. 3), and Period F, which saw significantly more importations to NI than RoI, may be significant.

Furthermore, Fig. 9C, D show that the decrease in genome cytosine (C) content (Fig. 9C) is generally compensated by an increase in genome thymine (T) content (Fig. 9D), which is characteristic of SARS-CoV-2 evolutionary behaviour reported previously [11, 12].

## Discussion

The unprecedented global sequencing and open sharing of SARS-CoV-2 samples created a rich resource for addressing previously intractable research questions [71, 127]. The work of many research groups to optimise phylogenetic analysis of millions of SARS-CoV-2 sequences provided the tools we required to exploit this huge dataset and identify introduction events to the island of Ireland during key periods of the pandemic [69, 128, 129].

Despite our extensive analyses, the 4403 independent SARS-CoV-2 introductions to Ireland detected during the six time periods studied should be treated as a conservative lower bound estimate for several reasons. Firstly, to identify independent introductions, it is necessary to distinguish them from each other. Genetic diversity facilitates the tracking of viral infection and onward transmission events for the purposes of genomic surveillance [130, 131]. Novel shared mutations reveal a history of infection and transmission events. Similarly, distinguishing between separate introduction events is only possible if they are genetically distinct. Identical sequences resulting from multiple introduction events would be grouped in the same cluster, leading them to be counted as a single introduction. The relatively low mutation rate and genetic diversity of SARS-CoV-2 contribute to an increased likelihood of such occurrences, resulting in an underestimation of independent introduction events.

Secondly, the Irish genome sequencing data is limited by sparseness and a variable sampling rate. In 2020, the rate of sequencing in both NI and RoI was low―with less than three thousand sequences from each, compared to approximately fifty thousand each in 2021. This restricts how informative earlier time periods, specifically A, B, and much of C, are for gleaning insights into Irish introductions. Key periods of importation that involve increased international travel and gatherings, such as Christmas, are also periods of reduced sampling. This has a twofold impact, increasing the rate of successful introductions taking place whilst simultaneously reducing the opportunity to capture these events. These factors within the Irish sequencing programmes directly affect the number of introductions observed and cause the extent of underestimation to vary.

Thirdly, the global inequality in SARS-CoV-2 genomic surveillance [132, 133] can influence the number of introductions identified. Consider a scenario in which a lineage with a distinctive combination of mutations, lineage L, is circulating in another country, country A. If a sample associated with country A is later sequenced in Ireland and exhibits the unique set of mutations in lineage L, it allows for easy attribution of country A as the origin. If a subsequent mutation, mutation M, occurs in country A and a mutated lineage, L_M_, is again introduced, it will be recognised as a second introduction event when sequenced in both countries. However, if the sequencing rate in country A is low, mutation M might only be sequenced amongst Irish samples. Therefore, mutation M would manifest as an Ireland-specific mutation within the initial introduction cluster of lineage L. While rare, this situation may lead to missed introductions and merged clusters that should be considered as independent. High sequencing rates in nearby Great Britain should reduce the occurrence of such scenarios affecting our introduction counts for Ireland.

A low sampling rate in other countries is also a well recognised source of misattributions of origin. If a cryptic lineage circulates in a country, country B, with a low rate of sequencing it is likely not to be sampled and would therefore not be attributed to country B. Introductions from country B to countries other than Ireland can also occur, some of which may have robust variant detection programmes, potentially leading to some introductions to Ireland from country B being wrongly attributed to one of these other countries. We address this issue by normalising the proportions of introductions to Ireland from each country by the proportion of samples from that country in the global tree. While this enables less biased comparisons between sources of introductions, anomalies can occur when assessing introductions from countries with very few sequences.

We also acknowledge the potential for biases in genomic surveillance sampling due to factors such as regional healthcare infrastructure, testing availability, and public health policies. It is also important to consider potential variations in healthcare-seeking behaviours across different demographic and socioeconomic groups. While substantial efforts were made to ensure widespread testing during the COVID-19 pandemic in Ireland including in deprived areas, systemic barriers may have influenced testing access [25] and thus sequencing. Future studies could explore these cultural dynamics further to enhance understanding of potential sampling biases in Ireland and elsewhere. Such biases could influence observed transmission patterns. It should also be noted that the travel volume data does not distinguish between direct international arrivals and those with layovers in Great Britain. This categorisation might somewhat inflate arrival numbers from England. However, this should not affect the prediction of introductions, which is based on phylogenetics derived from global sequencing data and GISAID metadata for the country of collection. Future studies with detailed travel metadata could further clarify the impact of travel routes on introduction rates.

Notwithstanding the caveats discussed above, we were able to discern patterns of introductions that cannot be solely explained by variations in national sequencing levels. As anticipated, for some time periods, there is a negative correlation between number of introductions and distance, presumably reflecting less travel from distant countries. During Period A, we observed 170 initial introductions in NI and RoI, while a study conducted in Scotland, with a population of approximately 5 million, identified 283 initial introductions [41]. In Portugal, which has a population of approximately 10 million, another study reported 277 early introductions [52]. Our observed rate of introductions is therefore consistent with other regions, considering variations in population size and study methodologies. The integration of geospatial and temporal metadata with the monophyletic sequences belonging to specific introduction and spreading events enabled us to develop epidemiological models and better understand pandemic dynamics. We further hypothesise that, given a sufficiently high level of accurate viral genome sequencing, spatiotemporal clusters of virus spread can be used to predict the presence of undetected introduction events.

Our approach can also be used to explore whether socioeconomic factors impact viral introductions and spread. The correlations observed between population density, deprivation, and introductions and spread of SARS-CoV-2 suggest roles for these factors in enhancing viral introductions and spread and corroborate previous associations of SARS-CoV-2 incidence with deprivation in Ireland [134], particularly in NI [25]. Targeted interventions in areas of high population density and deprivation may therefore be warranted to reduce the introductions and spread of SARS-CoV-2 and other future transmissable diseases. However, the relationship between deprivation and geographic location is complex; not all individuals experiencing deprivation live in areas typically associated with deprivation, and not all individuals living in such areas experience deprivation. This complexity underscores the need for further research to understand how deprivation and geographic factors interact to influence SARS-CoV-2 introduction and spreading rates. Thus, incorporating high-resolution geospatial data such as the 890 Super Output Areas [135] or 3780 Data Zones [136] in NI compared to the 11 LGDs used in this study, due to the limited resolution of GISAID metadata, could enhance the accuracy of analyses and provide a more nuanced understanding of the distribution of population density and deprivation and their impacts on viral introductions and spread.

The apparent distribution of SARS-CoV-2 infections follows a long-tailed distribution, with more than half of the clusters comprising only one Irish descendent. Although the size of most clusters will have been underestimated due to limited sampling, it is important to note that many clusters are small due to the infections being self-limited, with a relatively small number of super-spreading events fueling population-level incidence.

Attributing specific causes for differences between time periods is challenging due to the multiple factors that influence the number of observed introductions. For example, the relative scarcity of introductions observed in Period B is likely due to very limited travel but is also exacerbated by the low levels of sequencing in Ireland and globally. This raises the salient question: what level of sequencing is required to provide adequate genomic surveillance? Sequencing every single positive case will provide the most comprehensive representation of circulating variants in a community. However, this approach could be too costly and largely unnecessary since, in many cases, the same variants would be sampled repeatedly. Conversely, sequencing an insufficient number of cases poses the risk of missing variants of concern, whether imported or arising de novo. Such variants may remain undetected until they become well established in the community. To address this dilemma, we have developed a methodology that offers additional information to aid public health authorities in determining optimal sequencing levels: firstly, the number of sequences per capita required to estimate the total number of importations that are likely to have occurred and, secondly, the minimal number of sequences per capita required to detect all importations for a given period. Our approach maximises the use of available data to extrapolate from limited data points to gain valuable insights into viral transmission dynamics during these periods. Despite the lower sequencing rates in Periods A–C, we estimate the predicted number of sequences necessary to identify all additional uncaptured introduction events. The level of sequencing introduces variability in confidence levels, with higher confidence more probable in periods with more extensive sequencing coverage (D–F). Future research using larger and more diverse datasets will be essential to corroborate and expand upon our findings. Despite these methodological considerations, our approach offers a tool for understanding optimal sampling strategies rather than attempting to comprehensively map a complete transmission network.

The higher per capita introduction rate into NI during Period D (Delta) was likely due to higher travel into NI than RoI at this time (Fig. 2E). The similar COVID-19 incidence in both jurisdictions during Period D Fig. 2A) implies that most cases across Ireland arose due to internal spread and the benefit of travel restrictions was largely limited to reducing the risk of importing novel variants. In this scenario, the effect of new introductions can be likened to that of throwing additional matches into an already raging wildfire. It is difficult to attribute the small differences between RoI and NI in both the incidence (Fig. 2A) and rates of introductions (Fig. 6E) during the other periods to specific measures, which were broadly similar between jurisdictions [137, 138].

Viral sequencing of selected infected individuals makes it possible to investigate the relationships between viral characteristics (e.g. genetic identity and load) and clinical outcomes (e.g. symptoms, complications, disease severity, duration of illness, and treatment responses). When multiple individual sequences from a sufficiently large proportion of the population are available, as in this study, phylogenetic reconstructions can be used to track viral dynamics and identify outbreaks and chains of transmission events. An alternative approach to study population-level variant dynamics is the monitoring of viruses within wastewater using molecular methods. This provides unbiased aggregate sampling of entire communities and its effectiveness has already been demonstrated in Ireland [139, 140]. Despite the challenges of genome sequencing from wastewater, it has been shown to be an effective complementary approach for genomic surveillance of SARS-CoV-2 [141], although without the information that can be gained from the reconstruction of a molecular phylogeny.

In the context of infectious disease surveillance and outbreak response, phylogenetic analysis has already been shown to be a valuable tool [142] for identifying emerging strains [56], tracking the spread of disease [35, 52], and strategising targeted interventions [143]. Our study extends some of these applications and further advantages could be achieved by incorporating phylogenetic analyses into real-time advisory workflows [50, 96, 129]. This requires a robust system for collecting, storing, and analysing genetic sequence data as well as trained personnel who are skilled in bioinformatics and phylogenetic analyses [144]. By leveraging the power of genetic sequencing and analysis, public health agencies and other stakeholders can better understand the evolution and transmission of pathogens, identify at-risk populations, and develop effective strategies to prevent and control outbreaks. Increased use of phylogenetic analyses in real-time advisory workflows has the potential to significantly improve our ability to respond to infectious disease threats, ultimately leading to better public health outcomes.

## Conclusions

This analysis enhances the well recognised potential of genomic epidemiology [50, 145–147] despite relatively sparse and variable sequencing. The phylogeographic techniques used in this study allow (1) estimation of the origin and source of introductions, (2) mapping of subsequent spreading events and propagation, (3) estimation of substitution rates within introduction clusters, and (4) providing broader insights into the effects of viral lineage on transmission dynamics. Although our conclusions must be tempered by fluctuations in local and international sequencing, the estimates of numbers of introductions and geospatial tracking of specific clusters could be used to inform public health agencies how to limit the spread of health risks to neighbouring countries and to prevent unwarranted travel and trade restrictions. These data could also assist vaccine deployment strategies and implicitly estimate herd immunity thresholds [145]. The WHO International Health Regulations (2005) aim to prevent and respond to the international spread of disease ‘while avoiding unnecessary interference with international traffic and trade’ [148]. Performing this analysis closer to real time and on an international scale could inform policy about the effectiveness of border measures.

The opportunity provided by the continually decreasing cost of WGS should be used to adopt WGS more widely to monitor and geospatiotemporally investigate future infectious conditions. Sequencing all infected individuals is not presently feasible nor is this necessary to predict the total number of introductions to geographic regions of interest. Our findings demonstrate the extensive variation in sequencing requirements across the SARS-CoV-2 pandemic. We therefore recommend a flexible strategy for surveillance with the capability to surge sequencing above baseline rates as required by the contemporaneous conditions.

## Supplementary Information

Additional file 1: Supplementary materials for SARS-CoV-2 introductions to the island of Ireland: a phylogenetic and geospatiotemporal study of infection dynamics. This file includes supplementary figures, links to supplementary animation figures S15–S19 [125], and supplementary tablesas follows: Figure S1. Global SARS-CoV-2 phylogeny. Figure S2. Sample collection vs. time-tree date estimates. Figure S3. Comparison of introductions detected via maximum likelihood and parsimony. Figure S4. Bivariate choropleth maps and OLS of population density and deprivation in Ireland. Figure S5–S10. Pruned trees for Periods A–F. Figure S11–S12. SARS-CoV-2 introductions by period. Figure S13. Additional geospatial spread examples. Figure S14. OLS regression on substitution rates by major lineage. Figure S15–S19. link to animations depicting geospatiotemporal spread for exemplary SARS-CoV-2 introduction events [125]. Table S1. Sample metadata mapped to Irish local districts. Table S2–S7. Originating country frequencies of importations. Table S8–S13. Originating country frequenciesusing maximum likelihood estimation. Table S14. OLS regression statistics for substitution rate estimation.

## Data Availability

Introduction cluster attributions and sizes for each period and GISAID EPI ISL accession codes for all sequences analysed are provided within our Zenodo repository [151]. Genomic data used in this study can be accessed by GISAID users who accept the license agreement. A companion website, which includes video files S15–S19 showcasing visualisations of geospatial introduction and spreading events of SARS-CoV-2, accompanies this publication [125]. Our datasets associating population density and deprivation in Ireland to SARS-CoV-2 introductions and spread are also available [123].

## Abbreviations

A: Adenosine
APOBEC: Apolipoprotein B mRNA-editing catalytic polypeptide-like)
C: Cytosine
COG-UK: COVID-19 Genomics UK
COVID-19: Coronavirus disease 2019
CSO: Central Statistics Office
DELTRAN: delayed transformation
G: Guanine
GISAID: Global Initiative on Sharing All Influenza Data
GIS: Geographic Information System
GWR: Geographically weighted regression
HGV: Heavy Goods Vehicle
ID: Identifier
Lat: Latitude
LGD: Local government district
Lon: Longitude
NI: Northern Ireland
NISRA: Northern Ireland Statistics and Research Agency
OLS: Ordinary least-squares
OSNI: Ordnance Survey of Northern Ireland
RNA: Ribonucleic acid
RoI: Republic of Ireland
SARS-CoV-2: Severe acute respiratory syndrome coronavirus 2
SOA: Super Output Area
UK: United Kingdom
USA: United States of America
U: Uracil
WGS: Whole genome sequencing
WHO: World Health Organization

## Code availability

The analysis of data was conducted in Jupyter Notebook [152]; analysis notebooks used in this study can be accessed [150].
