# Supplementary material for: SARS-CoV-2 introductions to the island of Ireland: a phylogenetic and geospatiotemporal study of infection dynamics
Source: Genome Med. 2024 Dec 19;16:150. doi: 10.1186/s13073-024-01409-1 (PMC11658175; doi:10.1186/s13073-024-01409-1)
Supplement: Supplementary file 1 — Additional file 1: Supplementary materials for SARS-CoV-2 introductions to the island of Ireland: a phylogenetic and geospatiotemporal study of infection dynamics. This file includes supplementary figures, links to supplementary animation figures S15–S19 [125], and supplementary tablesas follows: Figure S1. Global SARS-CoV-2 phylogeny. Figure S2. Sample collection vs. time-tree date estimates. Figure S3. Comparison of introductions detected via maximum likelihood and parsimony. Figure S4. Bivariate choropleth maps and OLS of population density and deprivation in Ireland. Figure S5–S10. Pruned trees for Periods A–F. Figure S11–S12. SARS-CoV-2 introductions by period. Figure S13. Additional geospatial spread examples. Figure S14. OLS regression on substitution rates by major lineage. Figure S15–S19. link to animations depicting geospatiotemporal spread for exemplary SARS-CoV-2 introduction events [125]. Table S1. Sample metadata mapped to Irish local districts. Table S2–S7. Originating country frequencies of importations. Table S8–S13. Originating country frequenciesusing maximum likelihood estimation. Table S14. OLS regression statistics for substitution rate estimation. [file 13073_2024_1409_MOESM1_ESM.pdf]

Additional file 1: supplementary materials for SARS-CoV-2 introductions to the island of Ireland: a phylogenetic and geospatiotemporal study of infection dynamics

Alan M. Rice[1,†,‡], Evan P. Troendle[1,†], Stephen Bridgett[1], Behnam Firoozi Nejad[2], Jennifer M. McKinley[2], The COVID-19 Genomics UK consortium[3], National SARS-CoV-2 Surveillance & Whole Genome Sequencing (WGS) Programme[4], Declan T. Bradley[5,6], Derek Fairley[7], Connor G. G. Bamford[8], Timofey Skvortsov[9,*], and David A. Simpson[1,*]

[1] Wellcome–Wolfson Institute for Experimental Medicine, School of Medicine, Dentistry and Biomedical Sciences, Queen's University Belfast, Belfast, Northern Ireland, BT9 7BL, United Kingdom
[2] Geography, School of Natural and Built Environment, Queen's University Belfast, Belfast, Northern Ireland, BT7 1NN, United Kingdom
[3] The full list of all individual members and partners of COG-UK can be found at
`https://webarchive.nationalarchives.gov.uk/ukgwa/20230522152804/https://www.cogconsortium.uk/full-list-of-all-individual-members-and-partners-of-cog-uk`
[4] The list of affiliated partners and laboratories can be found at
`https://www.hpsc.ie/a-z/wholegenomesequencingsurveillanceprogramme`
[5] Public Health Agency, Belfast, Northern Ireland, BT2 8BS, United Kingdom
[6] Centre for Public Health, School of Medicine, Dentistry and Biomedical Sciences, Queen's University Belfast, Belfast, Northern Ireland, BT12 6BA, United Kingdom
[7] Regional Virus Laboratory, Belfast Health and Social Care Trust, Belfast, Northern Ireland, BT12 6BA, United Kingdom
[8] Institute for Global Food Security, School of Biological Sciences, Queen's University Belfast, Belfast, Northern Ireland, BT9 5DL, United Kingdom
[9] Medical Biology Centre, School of Pharmacy, Queen's University Belfast, Belfast, Northern Ireland, BT9 7BL, United Kingdom [†] The authors consider these individuals to be Joint First Authors.

[‡] Current address: UCD National Virus Reference Laboratory, University College Dublin, Belfield, Dublin 4, D04 E1W1, Ireland
[*] Correspondence to `t.skvortsov@qub.ac.uk` and `david.simpson@qub.ac.uk`

List of figures

Figure S1 – GISAID global SARS-CoV-2 phylogeny
Figure S2 – Sample collection date from metadata and their predicted date from time-tree estimate
Figure S3 – Comparison of detected introductions using maximum likelihood and maximum parsimony ancestral state reconstruction
Figure S4 – Period A pruned tree
Figure S5 – Period B pruned tree
Figure S6 – Period C pruned tree
Figure S7 – Period D pruned tree
Figure S8 – Period E pruned tree
Figure S9 – Period F pruned tree
Figure S10 – Bivariate sequential choropleth maps (9-class) and OLS relating population density and deprivation in Ireland
Figure S11 – SARS-CoV-2 introductions for Periods A, B, and C
Figure S12 – SARS-CoV-2 introductions for Periods D, E, and F
Figure S13 – Additional examples of geospatial spreading of introduced SARS-CoV-2 infection clusters
Figure S14 – OLS regressions of substitutions in Irish SARS-CoV-2 sequences over time per major introduction lineage
Figures S15–S19 – Animations depicting geospatiotemporal spread for exemplary SARS-CoV-2 introduction events can be found here.

List of tables

Table S1 – Metadata for mapping samples to local government districts in Ireland (NI and RoI)
Table S2 – Originating countries and their frequencies of importations for Period A

Table S3 – Originating countries and their frequencies of importations for Period B
Table S4 – Originating countries and their frequencies of importations for Period C
Table S5 – Originating countries and their frequencies of importations for Period D
Table S6 – Originating countries and their frequencies of importations for Period E
Table S7 – Originating countries and their frequencies of importations for Period F
Table S8 – Originating countries and their frequencies of importations for Period A using maximum likelihood estimation
Table S9 – Originating countries and their frequencies of importations for Period B using maximum likelihood estimation
Table S10 – Originating countries and their frequencies of importations for Period C using maximum likelihood estimation
Table S11 – Originating countries and their frequencies of importations for Period D using maximum likelihood estimation
Table S12 – Originating countries and their frequencies of importations for Period E using maximum likelihood estimation
Table S13 – Originating countries and their frequencies of importations for Period F using maximum likelihood estimation
Table S14 – OLS linear regression statistics for estimating substitution rates of major imported lineages to Ireland

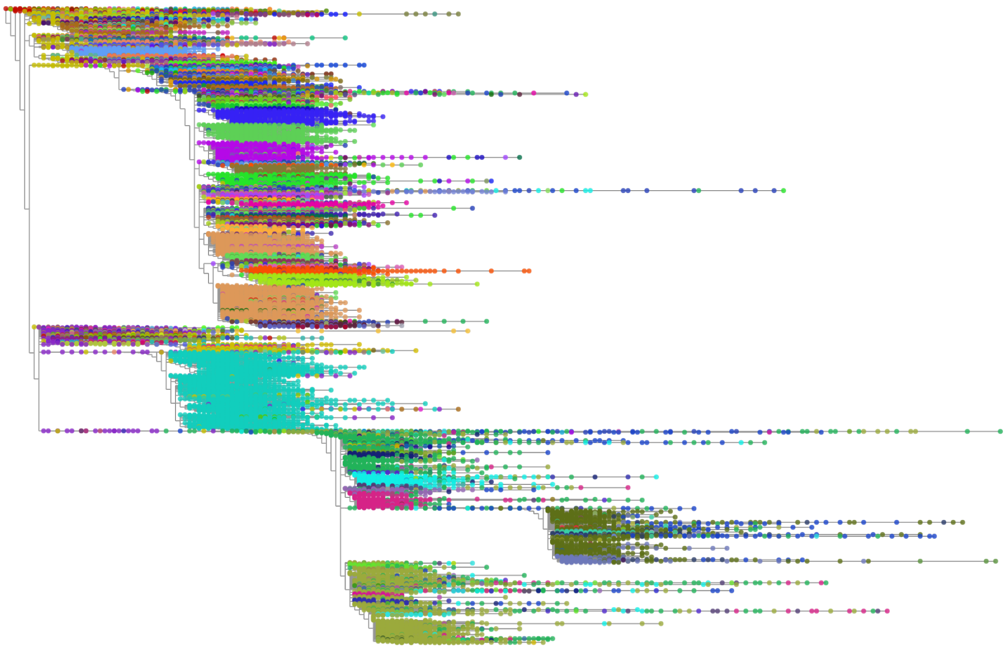

Figure S1. GISAID global SARS-CoV-2 phylogeny Global SARS-CoV-2 phylogeny, dated 20th May 2022, from GISAID of 7,603,547 SARS-CoV-2 genome sequences. Samples coloured by Pango [1] lineage and visualised using Taxonium [2].

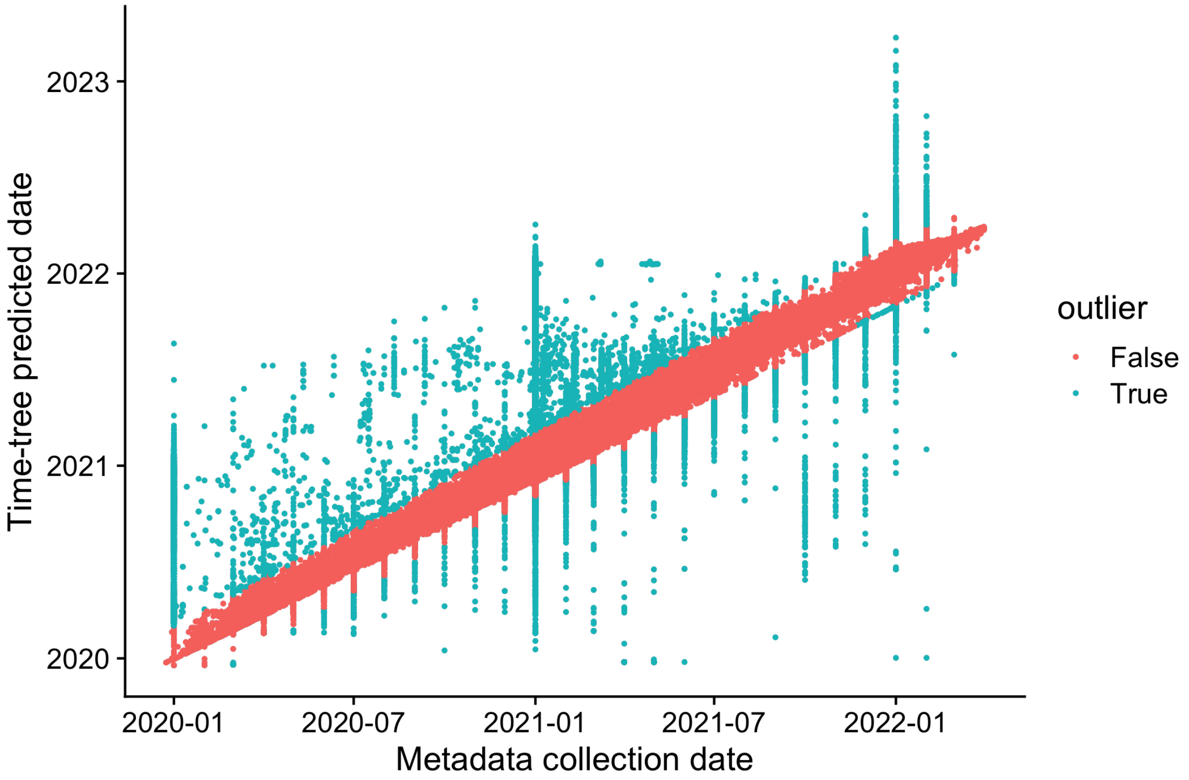

Figure S2. Sample collection date from metadata and their predicted date from time-tree estimate Sample collection date from GISAID metadata of sequences in phylogenetic tree and their predicted date from Chronumental time-tree estimate. Sequences that have a difference between metadata date and predicted date of z-score greater $\pm$ 3 are highlighted here as outliers.

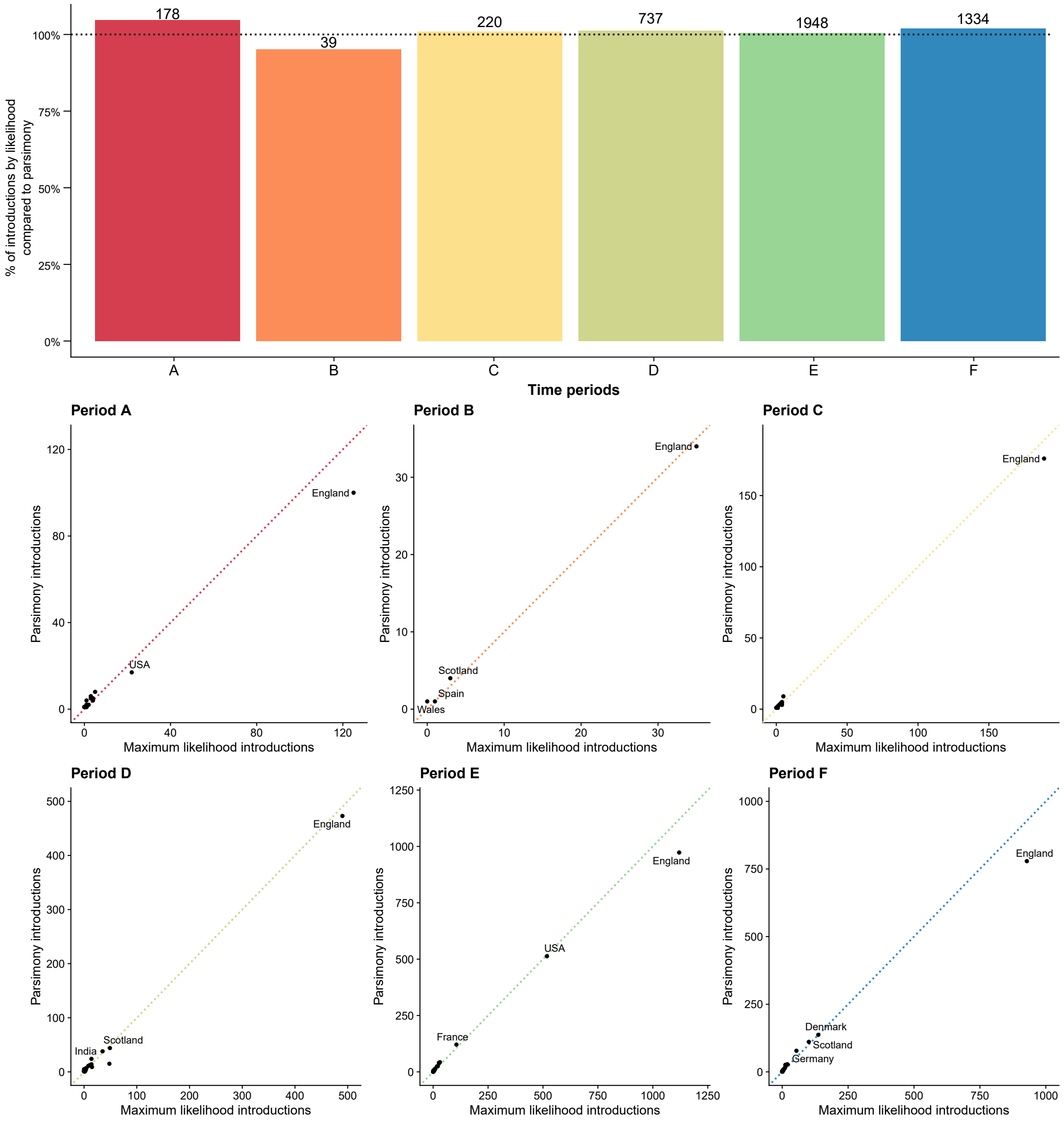

Figure S3. Comparison of detected introductions using maximum likelihood and maximum parsimony ancestral state reconstruction Top: The relative number of detected introductions per period using maximum likelihood (ML) by TreeTime ancestral state reconstruction compared to maximum parsimony (MP) using Delayed Transformation (DELTRAN). Bottom: For each period, the number of introductions to Ireland is broken down by suspected origin. The diagonal dotted lines ($y = x$) denote parity between assignments by ML and MP.

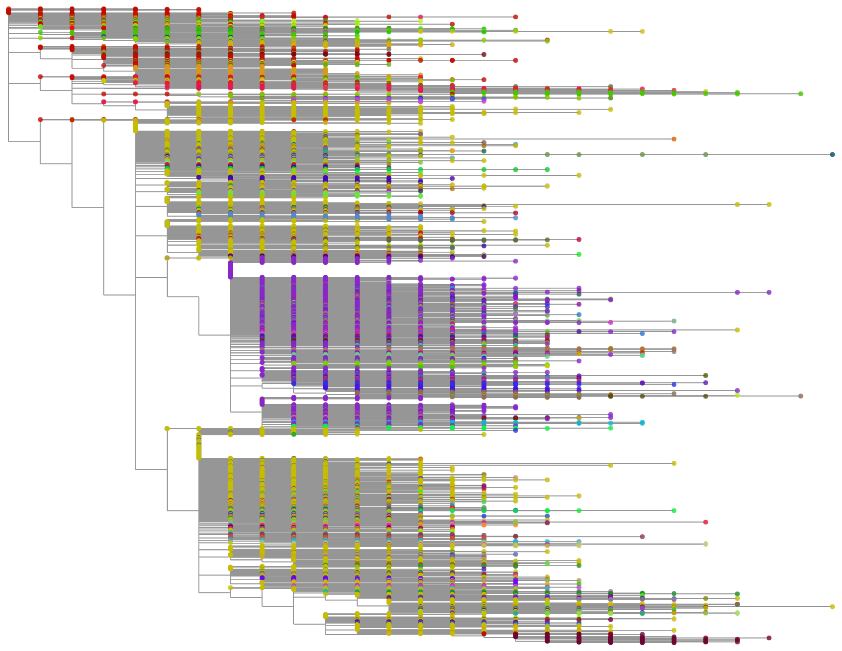

Figure S4. Period A pruned tree. Pruned SARS-CoV-2 phylogeny of 103,316 samples used for Period A analysis. Samples coloured by Pango [1] lineage and visualised using Taxonium [2].

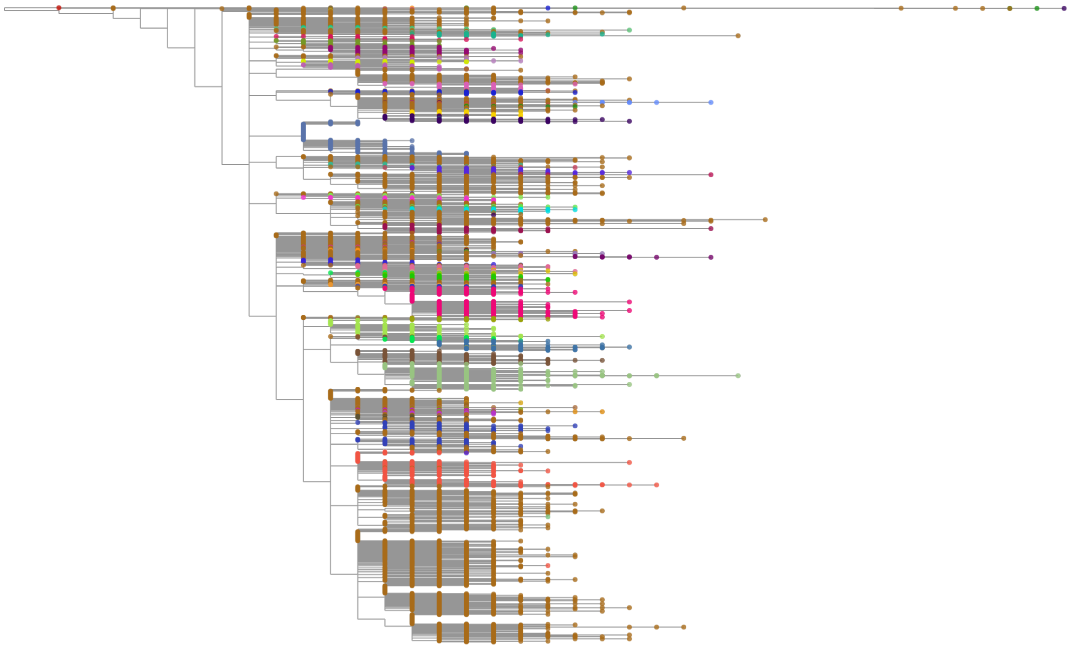

Figure S5. Period B pruned tree. Pruned SARS-CoV-2 phylogeny of 31,930 samples used for Period B analysis. Samples coloured by Pango [1] lineage and visualised using Taxonium [2].

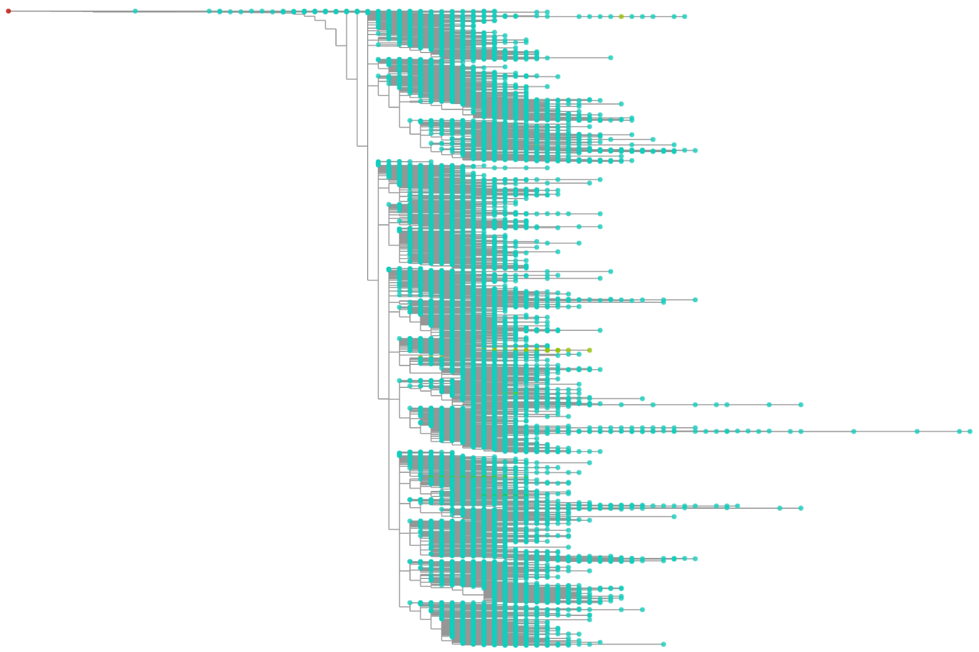

Figure S6. Period C pruned tree. Pruned SARS-CoV-2 phylogeny of 184,326 samples used for Period C analysis. Samples coloured by Pango [1] lineage and visualised using Taxonium [2].

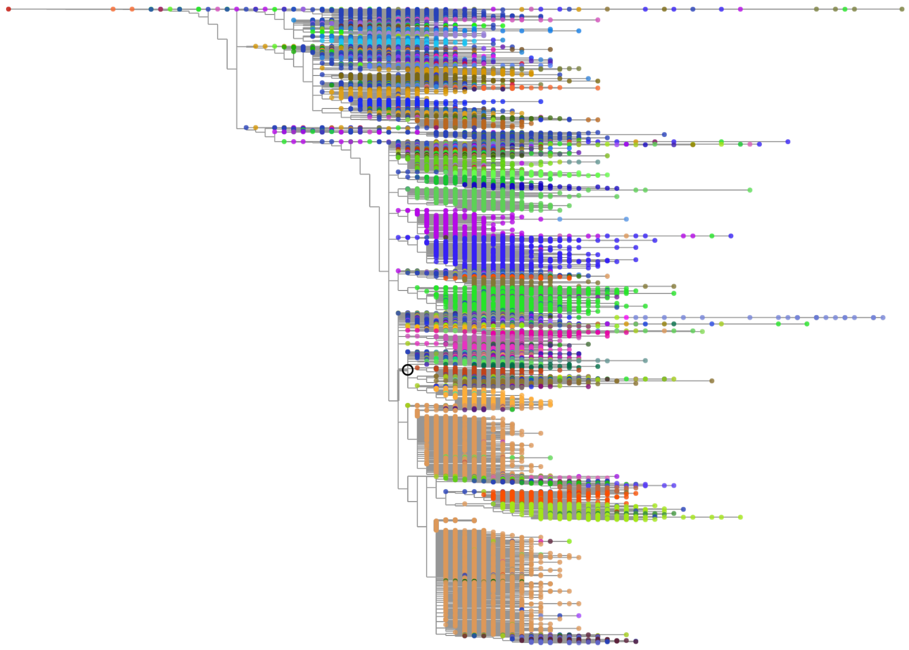

Figure S7. Period D pruned tree. Pruned SARS-CoV-2 phylogeny of 513,954 samples used for Period D analysis. Samples coloured by Pango [1] lineage and visualised using Taxonium [2].

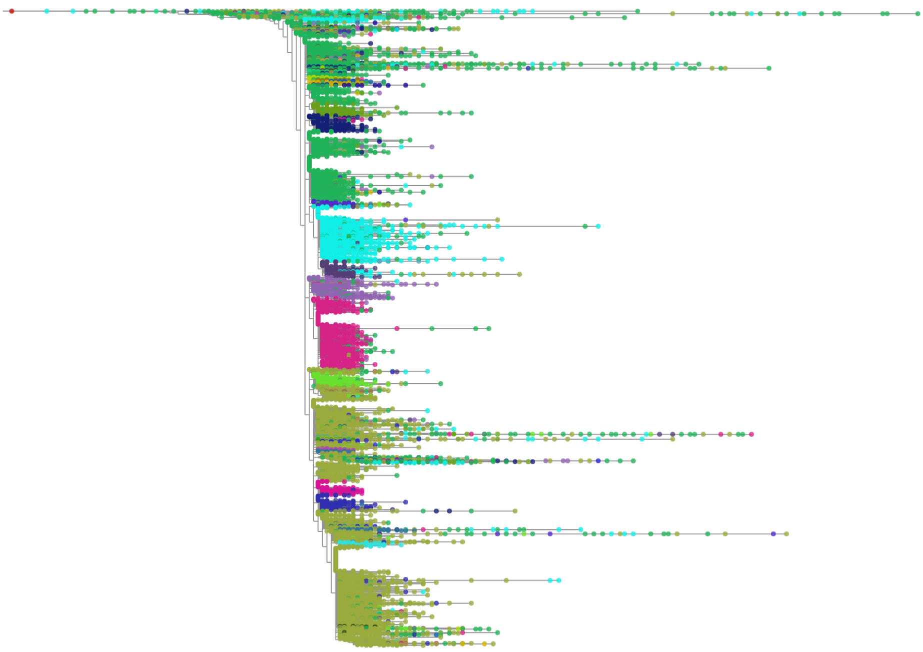

Figure S8. Period E pruned tree. Pruned SARS-CoV-2 phylogeny of 1,313,968 samples used for Period E analysis. Samples coloured by Pango [1] lineage and visualised using Taxonium [2].

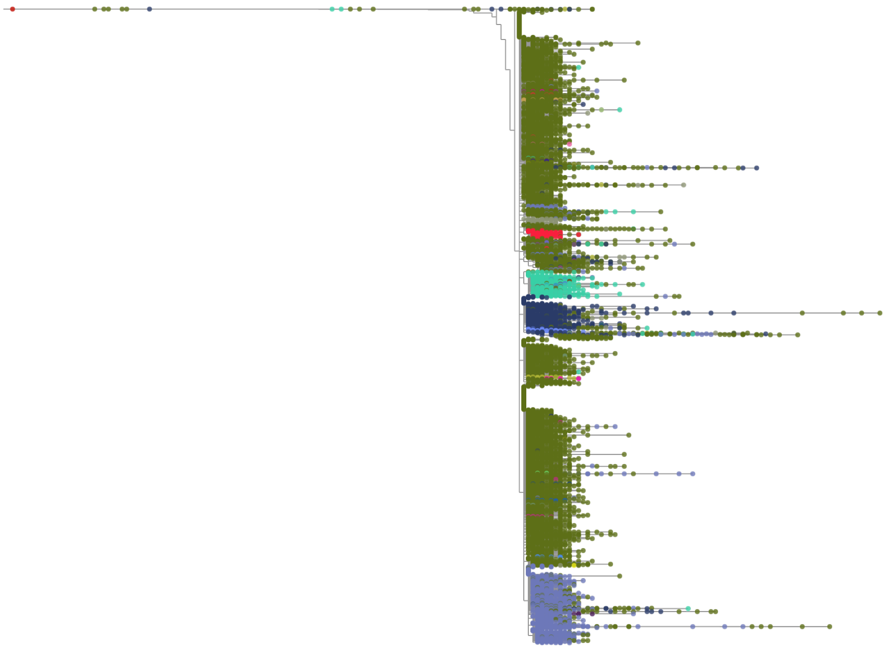

Figure S9. Period F pruned tree. Pruned SARS-CoV-2 phylogeny of 644,944 samples used for Period F analysis. Samples coloured by Pango [1] lineage and visualised using Taxonium [2].

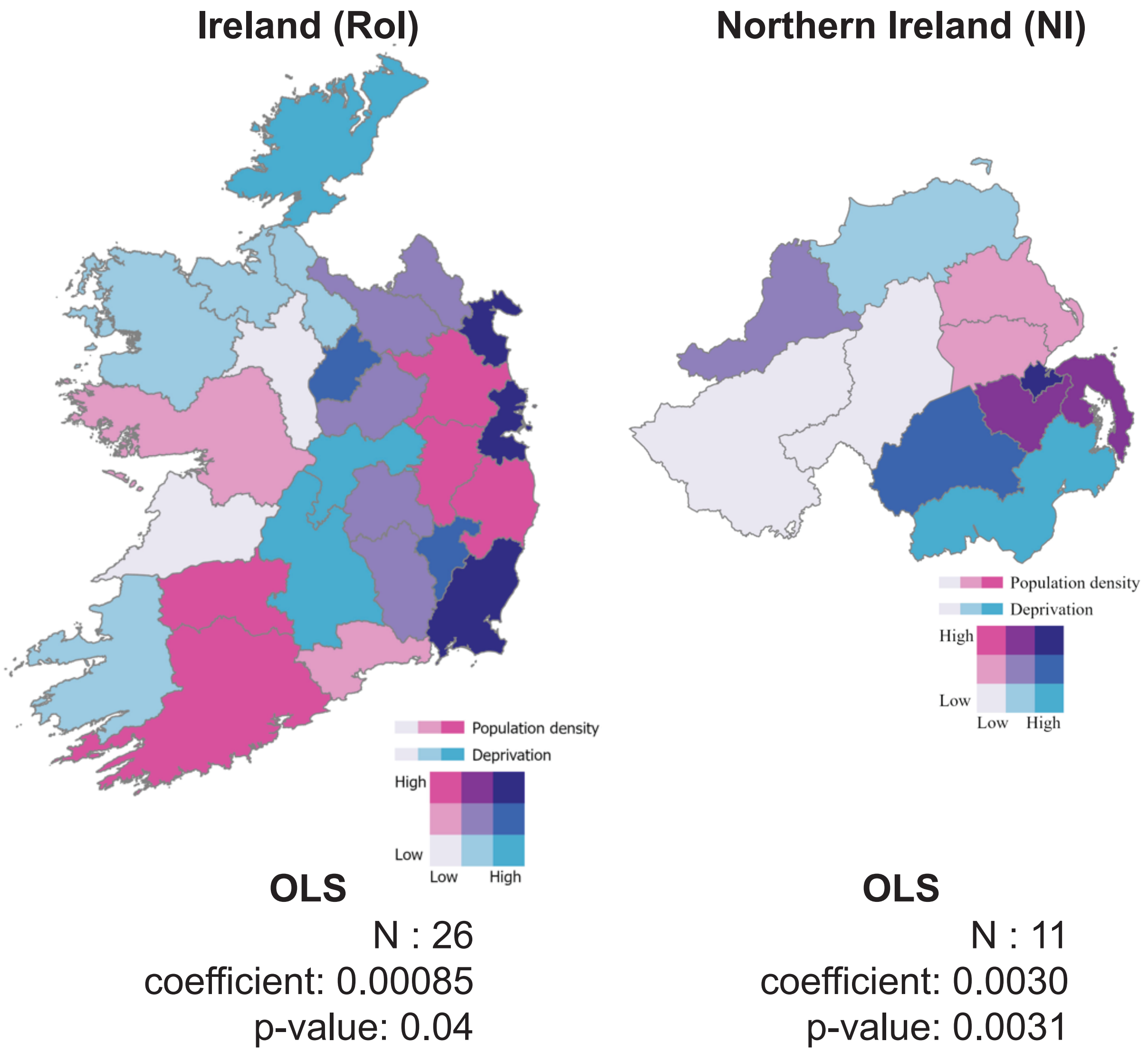

Figure S10. Bivariate sequential choropleth maps (9-class) and OLS relating population density and deprivation in Ireland

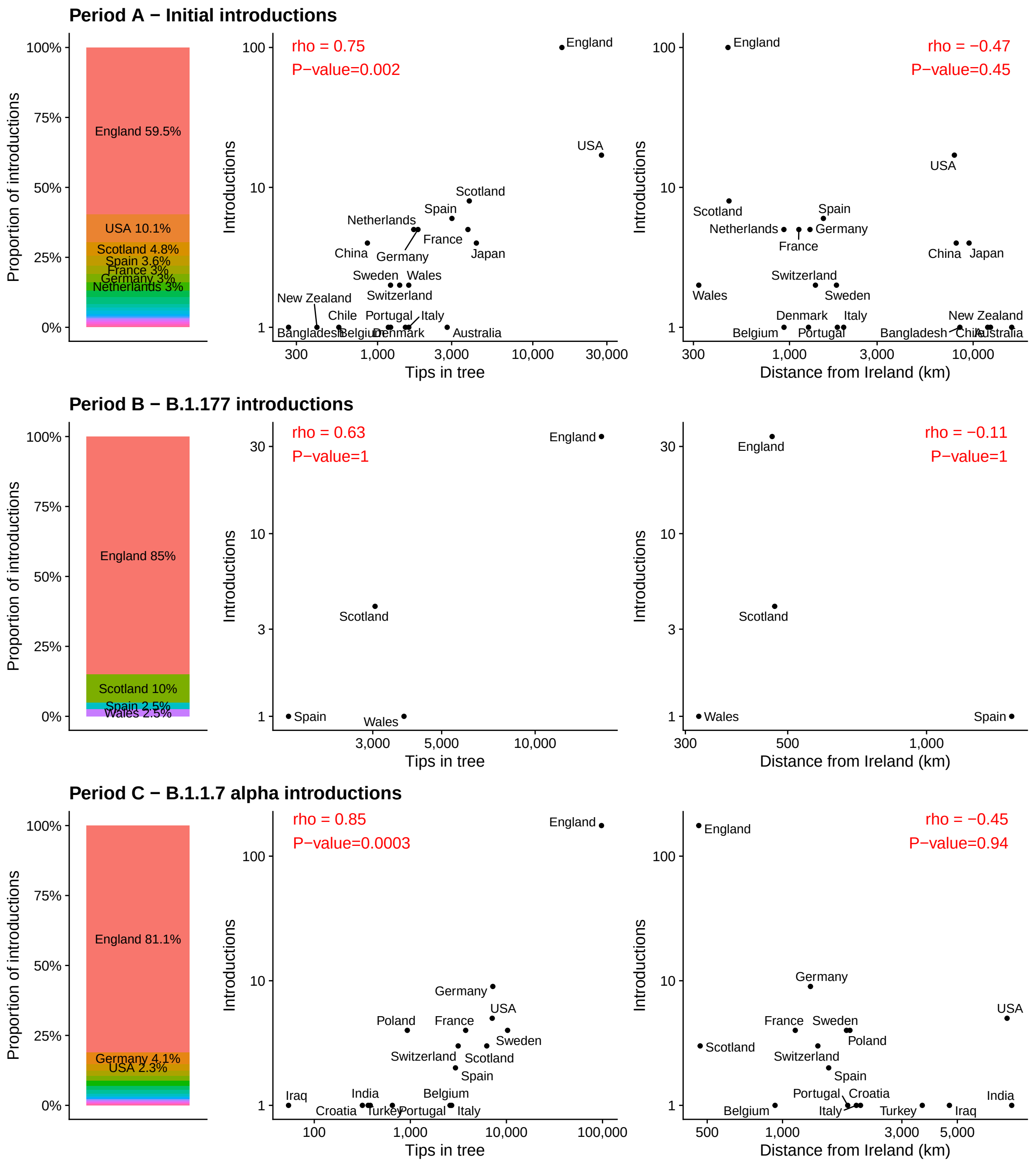

Figure S11. SARS-CoV-2 introductions for Periods A, B, and C. Left for each period, the proportion of importations from each country. Centre, the number of introductions and the number of tips per country in the tree. Right, the number of introductions per country and the distance in kilometres between Ireland and each respective country. Note the $\log_{10}$ scale for both the x and y axis. P-values for Spearman correlation tests are Bonferroni-corrected for multiple testing.

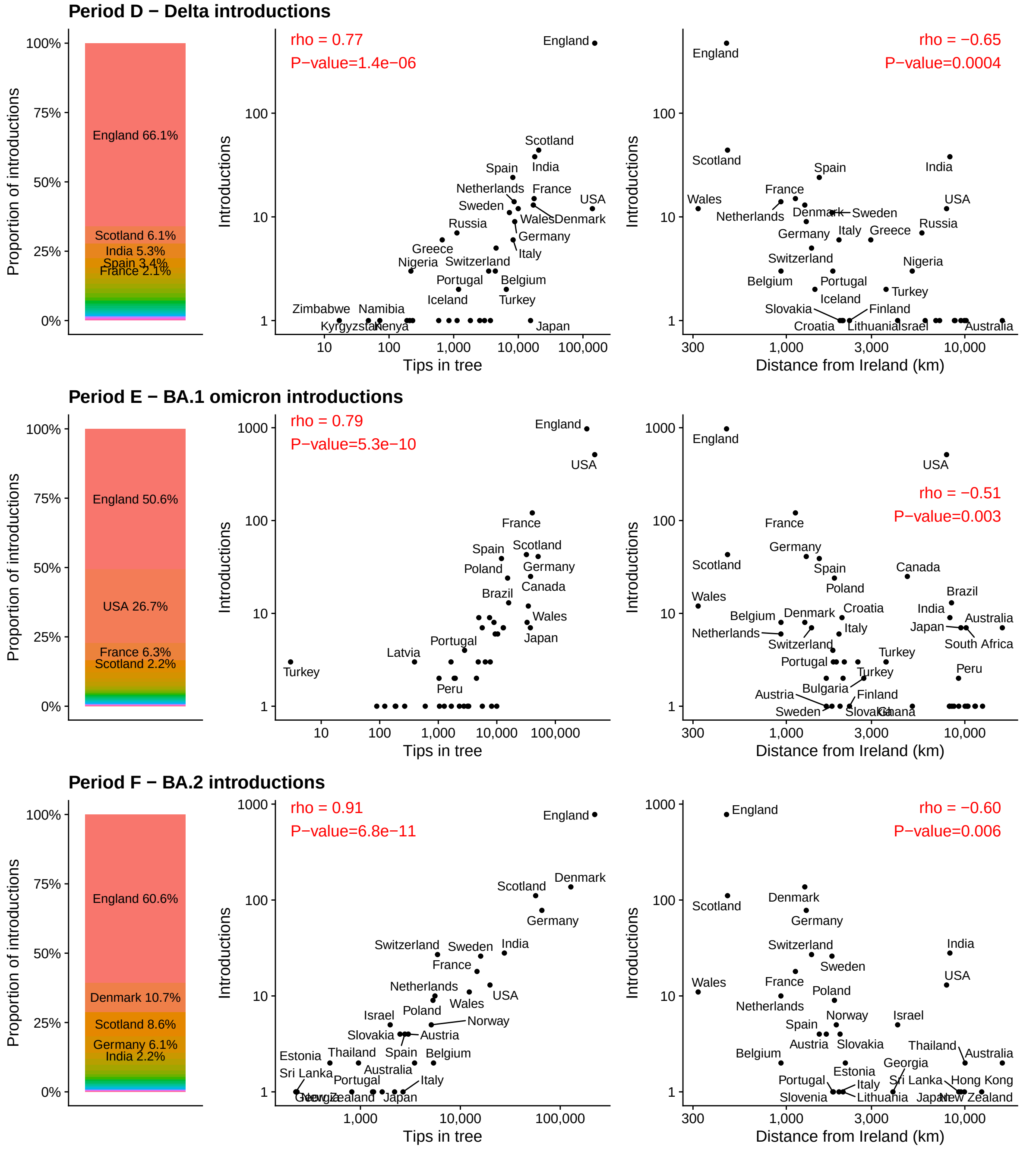

Figure S12. SARS-CoV-2 introductions for Periods D, E, and F. Left for each period, the proportion of importations from each country. Centre, the number of introductions and the number of tips per country in the tree. Right, the number of introductions per country and the distance in kilometres between Ireland and each respective country. Note the $\log_{10}$ scale for both the x and y axis. P-values for Spearman correlation tests are Bonferroni-corrected for multiple testing.

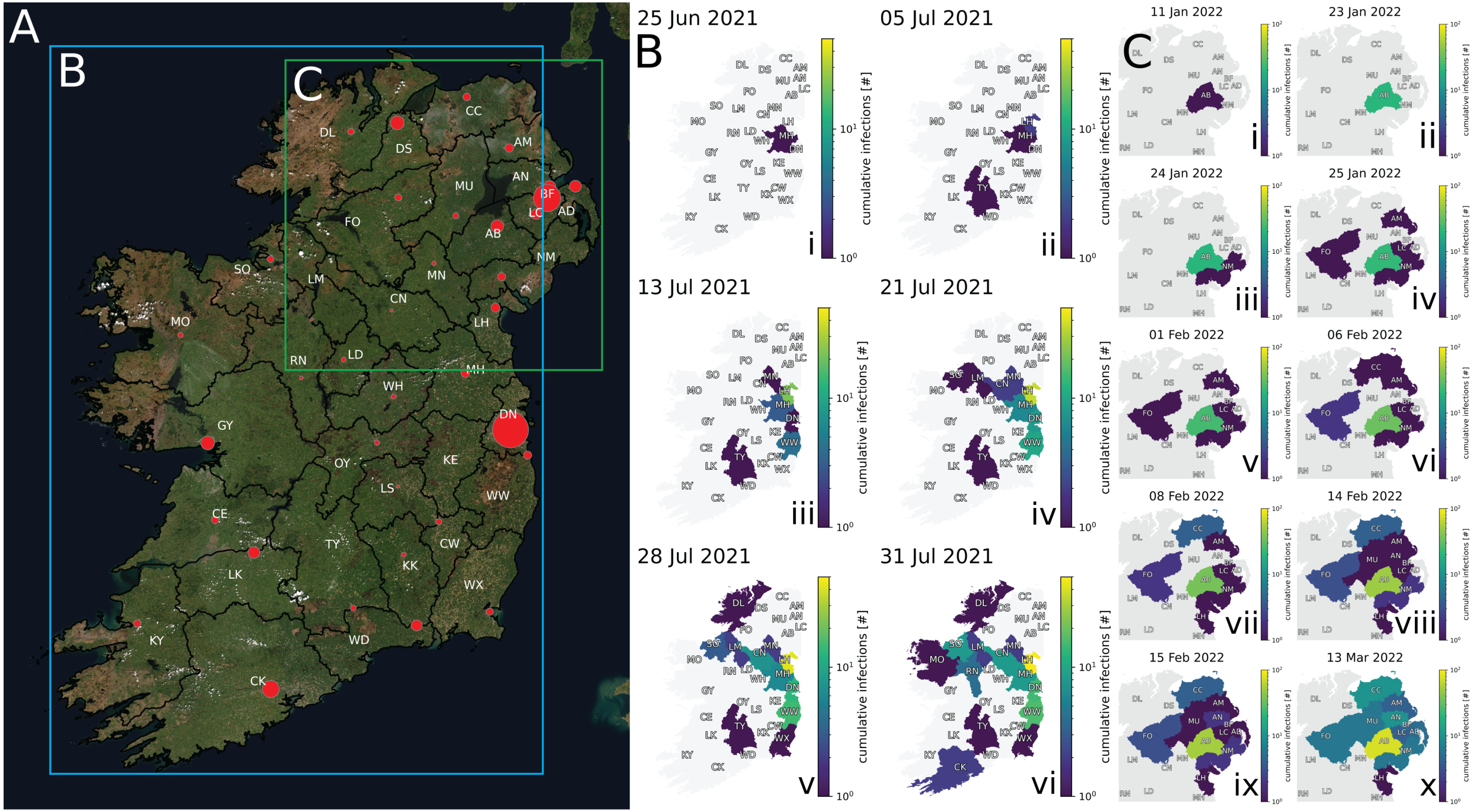

Figure S13. Geospatial tracking of the spread of samples from an introduction event within Ireland A, A map of Ireland created with GeoPandas v0.11.1 ([3], `https://geopandas.org/`) using the Esri "World Imagery" basemap (Sources: Esri, DigitalGlobe, GeoEye, i-cubed, USDA FSA, USGS, AEX, Getmapping, Aerogrid, IGN, IGP, swisstopo, and the GIS User Community) as retrieved using contextily v1.2.0 (`https://github.com/geopandas/contextily`). Red points designate the locations of population centres in each government district with the size of each point scaled according to recent population estimates. Refer to Table S1 for the corresponding metadata for region abbreviations and population centres. Bounding boxes B (cyan) and C (green) depict the geographic extents of the two largest introduction clusters detected in this study, which are elucidated to the right. (caption continues on the following page.)

Figure S13. Geospatial tracking of the spread of samples from an introduction event within Ireland (continued) B-i, An introduction of Delta SARS-CoV-2 descended from England begins an introduction cluster in County Meath in RoI on 25th June 2021. **B-ii**, The introduction cluster exhibits a non-neighbouring spreading event into County Tipperary as well as adjacently to County Louth by 5th July 2021. **B-iii**, By 13th July 2021, the infection cluster has adjacently spread to County Monaghan, County Dublin, and County Waterford. **B-iv**, The introduction cluster reaches County Leitrim and County Sligo by 21st July 2021. **B-v**, County Donegal and County Wexford are reached by the introduction cluster by 28th July 2021. **B-vi**, The final geographic extent of the introduction cluster is observed, having reached County Cork, County Mayo, and County Roscommon by 31th July 2021. **C-i**, An introduction of BA.2 SARS-CoV-2 descended from Scotland is detected within the boundaries of Armagh City, Banbridge and Craigavon in NI on 11th January 2022. **C-ii**, 20 more infections afflict other individuals within the jurisdictional boundaries of Armagh City, Banbridge and Craigavon in NI through 23 January 2022. **C-iii**, An individual within the Newry, Mourne and Down is infected on 24th January 2022. **C-iv**, the infection cluster reaches three new NI local government districts, namely Fermanagh and Omagh, Mid and East Antrim, and Lisburn and Castlereagh on 25th January 2022. **C-v**, An affliction in Belfast belonging to the cluster is noted on 1st February 2022 **C-vi**, Spread of this importation cluster reaches Causeway Coast and Glens within on 6th February 2022. **C-vii**, The cluster reaches RoI by afflicting an individual in County Louth on 8th February 2022. **C-viii**, Further spreading occurs to Antrim and Newtownabbey and Mid Ulster **C-ix**, The clustered infection spread to its final geographic coverage having reached Ards and North Down on 15th February 2022 **C-x**, Spreading within the aforementioned regions continues until the last sequenced case in the cluster was recorded on 13th Mar 2022, indicating that this introduction cluster was tracked over 61 days.

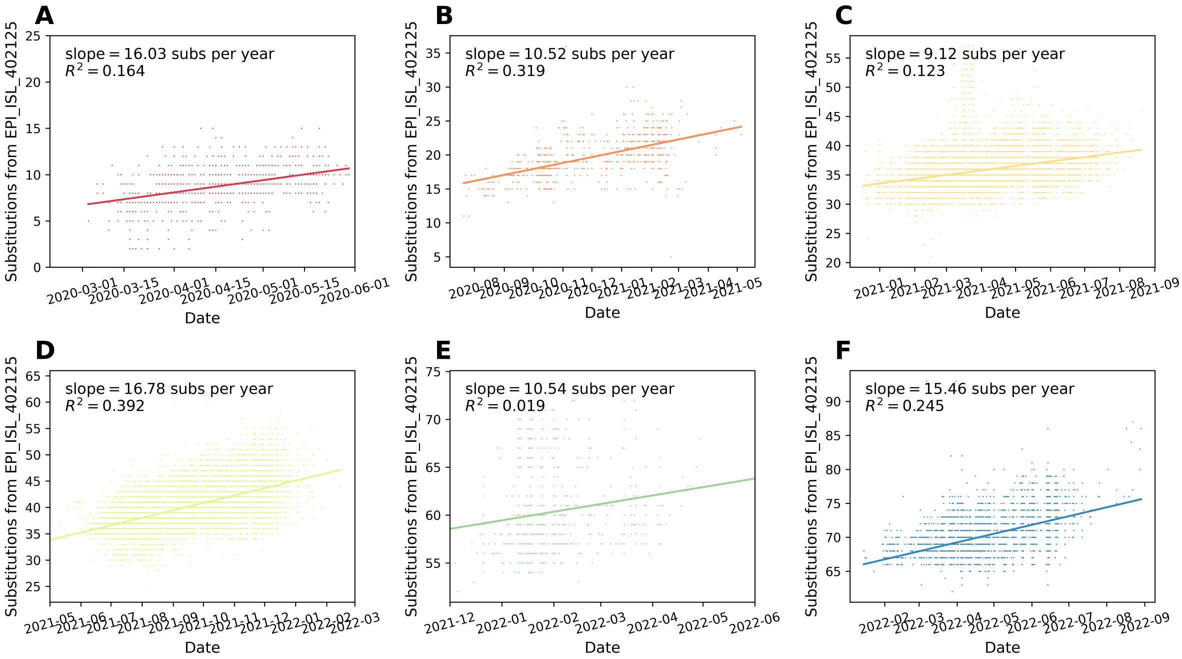

Figure S14. OLS regressions of substitutions in Irish SARS-CoV-2 sequences over time per major introduction lineage

Genome coverage for all samples included were at least 99.9% upon alignment to the Wuhan-Hu-1 SARS-CoV-2 reference (GISAID: EPI_ISL_402125). See Table S14 for related statistics. **A**, Initial introductions (Period A) **B**, B.1.177 (Period B) **C**, B.1.1.7 (Period C) **D**, Delta (Period D) **E**, Omicron (BA.1*) (Period E) **F**, Omicron (BA.2*) (Period F)

Table S1. Metadata for mapping samples to local government districts in Ireland (NI and RoI) Population data within NI and RoI were obtained from NISRA [4] and CSO [5], respectively. Troendle–Rice–Simpson–Skvortsov 2-letter abbreviation codes were devised for each local government district.

Country	Local government district	Abbreviation	Area [km^2]	Population	Population centre (PC)	PC Population	PC Latitude	PC Longitude
NI	Antrim and Newtownabbey	AN	571	145661	Newtownabbey	67200	54.6570°N	5.9070°W
NI	Ards and North Down	AD	457	163659	Bangor	63480	54.6600°N	5.6700°W
NI	Armagh City, Banbridge and Craigavon	AB	1347	218656	Craigavon	71560	54.4472°N	6.3883°W
NI	Belfast	BF	134	345418	Belfast	337210	54.5973°N	5.9301°W
NI	Causeway Coast and Glens	CC	1980	141746	Coleraine	25540	55.1320°N	6.6685°W
NI	Derry City and Strabane	DS	1237	150756	Derry/Londonderry	85080	54.9958°N	7.3074°W
NI	Fermanagh and Omagh	FO	2847	116812	Omagh	20000	54.6003°N	7.2984°W
NI	Lisburn and Castlereagh	LC	503	149106	Lisburn	49890	54.5120°N	6.0310°W
NI	Mid and East Antrim	AM	1046	138994	Ballymena	31270	54.8631°N	6.2783°W
NI	Mid Ulster	MU	1821	150293	Dungannon	15860	54.5029°N	6.7696°W
NI	Newry, Mourne and Down	NM	1619	182074	Newry	28410	54.1760°N	6.3490°W
RoI	Carlow	CW	895	61931	Carlow	16200	52.8360°N	6.9245°W
RoI	Cavan	CN	1856	81201	Cavan	3805	53.9943°N	7.3608°W
RoI	Clare	CE	3159	127419	Ennis	22100	52.8463°N	8.9807°W
RoI	Cork	CK	7255	581231	Cork	130119	51.8972°N	8.4700°W
RoI	Donegal	DL	4764	166321	Letterkenny	130119	54.9490°N	7.7342°W
RoI	Dublin	DN	924	1450701	Dublin	588233	53.3441°N	6.2675°W
RoI	Galway	GY	5796	276451	Galway	83456	53.2719°N	9.0489°W
RoI	Kerry	KY	4679	155528	Tralee	22300	52.2675°N	9.6962°W
RoI	Kildare	KE	1693	246977	Newbridge	7767	53.1805°N	6.7959°W
RoI	Kilkenny	KK	2061	103685	Kilkenny	9729	52.6537°N	7.2480°W
RoI	Laois	LS	1719	91657	Portlaoise	3920	53.0309°N	7.3008°W
RoI	Leitrim	LM	1502	35087	Carrick-on-Shannon	4062	53.9440°N	8.0950°W
RoI	Limerick	LK	2683	205444	Limerick	61570	52.6653°N	8.6238°W
RoI	Longford	LD	1040	46634	Longford	9600	53.7270°N	7.7998°W
RoI	Louth	LH	824	139100	Dundalk	35300	54.0090°N	6.4049°W
RoI	Mayo	MO	5351	137231	Castlebar	12068	53.8608°N	9.2988°W
RoI	Meath	MH	2332	220296	Navan	31800	53.6528°N	6.6814°W
RoI	Monaghan	MN	1273	64832	Monaghan	7678	54.2479°N	6.9708°W
RoI	Offaly	OY	1995	82668	Tullamore	11894	53.2739°N	7.4945°W
RoI	Roscommon	RN	2445	69995	Roscommon	5876	53.6279°N	8.1886°W
RoI	Sligo	SO	1791	69819	Sligo	18518	54.2706°N	8.4716°W
RoI	Tipperary	TY	4248	167661	Clonmel	14800	52.3539°N	7.7116°W
RoI	Waterford	WD	1836	127085	Waterford	54352	52.2567°N	7.1292°W
RoI	Westmeath	WH	1756	95840	Mullingar	10732	53.5258°N	7.3412°W
RoI	Wexford	WX	2353	163527	Wexford	22200	52.3342°N	6.4575°W
RoI	Wicklow	WW	2000	155485	Bray	28400	53.2044°N	6.1092°W

Table S2. Originating countries and their frequencies of importations for Period A. Rows in italics could not be fully resolved and are ambiguous.

Origin country	Both	Into RoI	Into NI
Republic of Ireland (RoI)	–	–	2
Northern Ireland (NI)	–	1	–
England	100	61	39
USA	17	12	5
Scotland	8	1	7
Spain	6	4	2
France	5	3	2
Germany	5	3	2
Netherlands	5	4	1
China	4	3	1
Japan	4	2	2
Sweden	2	1	1
Switzerland	2	1	1
Wales	2	1	1
Portugal	1	1	0
Italy	1	1	0
Belgium	1	1	0
Chile	1	0	1
Bangladesh	1	1	0
Australia	1	0	1
New Zealand	1	1	0
Denmark	1	1	0
Chile / RoI / England	1	1	0
Germany / Austria	1	0	1
**Total into Ireland**	170	103	67

Table S3. Originating countries and their frequencies of importations for Period B. Rows in italics could not be fully resolved and are ambiguous.

Origin country	Both	Into RoI	Into NI
Republic of Ireland (RoI)	–	–	0
Northern Ireland (NI)	–	0	–
England	34	13	21
Scotland	4	3	1
Wales	1	0	1
Spain	1	1	0
Latvia / Norway / Lithuania / Iceland	1	1	0
**Total into Ireland**	41	18	23

Table S4. Originating countries and their frequencies of importations for Period C. Rows in italics could not be fully resolved and are ambiguous.

Origin country	Both	Into RoI	Into NI
Republic of Ireland (RoI)	–	–	11
Northern Ireland (NI)	–	14	–
England	176	137	39
Germany	9	6	3
USA	5	5	0
Poland	4	3	1
France	4	4	0
Sweden	4	4	0
Switzerland	3	3	0
Scotland	3	2	1
Spain	2	2	0
Turkey	1	1	0
Belgium	1	1	0
India	1	1	0
Croatia	1	1	0
Italy	1	1	0
Portugal	1	1	0
Iraq	1	1	0
Germany / England	1	0	1
**Total into Ireland**	218	173	45

Table S5. Originating countries and their frequencies of importations for Period D. Rows in italics could not be fully resolved and are ambiguous.

Origin country	Both	Into RoI	Into NI	*Into RoI/NI*
Republic of Ireland (RoI)	–	–	42	
Northern Ireland (NI)	–	31	–	
England	473	244	228	1
Scotland	44	19	25	
India	38	33	5	
Spain	24	23	1	
France	15	15	0	
Netherlands	14	14	0	
Denmark	13	12	1	
USA	12	12	0	
Wales	12	4	8	
Sweden	11	11	0	
Germany	9	9	0	
Russia	7	6	1	
Greece	6	6	0	
Italy	6	5	1	
Switzerland	5	5	0	
Belgium	3	3	0	
Portugal	3	2	1	
Nigeria	3	3	0	
Turkey	2	2	0	
Iceland	2	2	0	
Kenya	1	1	0	
Lithuania	1	1	0	
Hong Kong	1	1	0	
Australia	1	1	0	
Japan	1	1	0	
Uganda	1	1	0	
Namibia	1	1	0	
Wales—England	1	0	1	
Kyrgyzstan	1	1	0	
Croatia	1	1	0	
Israel	1	1	0	
Zimbabwe	1	1	0	
South Africa	1	1	0	
Slovakia	1	1	0	
Finland	1	1	0	
England / RoI	4	4	0	
Italy / Belgium	1	1	0	
Netherlands / Denmark / RoI	1	1	0	
France / Belgium	1	1	0	
Italy / Denmark	1	1	0	
Germany / Israel	1	1	0	
Scotland / NI	1	0	1	
RoI / Portugal	1	1	0	
**Total into Ireland**	728	454	273	1

Table S6. Originating countries and their frequencies of importations for Period E. Rows in italics could not be fully resolved and are ambiguous.

Origin country	Both	Into RoI	Into NI
Republic of Ireland (RoI)	–	–	38
Northern Ireland (NI)	–	31	–
England	973	591	382
USA	513	379	134
France	121	105	16
Scotland	43	14	29
Germany	41	32	9
Spain	39	34	5
Canada	25	21	4
Poland	24	21	3
Brazil	13	10	3
Wales	12	7	5
Croatia	9	8	1
India	9	5	4
Belgium	8	8	0
Denmark	8	8	0
Switzerland	7	7	0
Japan	7	5	2
Australia	7	7	0
South Africa	7	4	3
Netherlands	6	6	0
Italy	6	4	2
Portugal	4	3	1
Turkey	3	3	0
Romania	3	2	1
Slovenia	3	2	1
Latvia	3	3	0
Norway	3	3	0
Peru	2	2	0
Czech Republic	2	2	0
Lithuania	2	2	0
Bulgaria	2	1	1
Slovakia	1	0	1
Bangladesh	1	1	0
Sri Lanka	1	1	0
Mauritius	1	1	0
Ghana	1	1	0
Mexico	1	1	0
Finland	1	1	0
Sweden	1	1	0
Malaysia	1	1	0
Costa Rica	1	1	0
Seychelles	1	1	0
Argentina	1	1	0
Indonesia	1	1	0
Thailand	1	1	0
Austria	1	1	0
Reunion	1	1	0
RoI / Poland	2	2	0
RoI / Germany	2	2	0
England / RoI	2	2	0
RoI / Switzerland	1	1	0
Netherlands / Poland	1	1	0
Scotland / NI	1	0	1
South Africa / Poland	1	1	0
RoI / Indonesia	1	1	0
France / Germany	1	1	0
RoI / Slovakia	1	1	0
Italy / Poland	1	1	0
Slovakia / Poland	1	1	0
RoI / USA	1	1	0
**Total into Ireland**	1,937	1,329	608

Table S7. Originating countries and their frequencies of importations for Period F. Rows in italics could not be fully resolved and are ambiguous.

Origin country	Both	Into RoI	Into NI
Republic of Ireland (RoI)	–	–	5
Northern Ireland (NI)	–	29	–
England	779	218	561
Denmark	137	41	96
Scotland	111	22	89
Germany	78	30	48
India	28	16	12
Switzerland	27	24	3
Sweden	26	9	17
France	18	9	9
USA	13	5	8
Wales	11	6	5
Netherlands	10	7	3
Poland	9	4	5
Israel	5	3	2
Norway	5	0	5
Austria	4	2	2
Spain	4	2	2
Slovakia	4	4	0
Belgium	2	2	0
Thailand	2	1	1
Australia	2	1	1
Estonia	2	0	2
Hong Kong	1	0	1
Sri Lanka	1	1	0
Slovenia	1	1	0
Japan	1	1	0
Portugal	1	1	0
Georgia	1	1	0
New Zealand	1	0	1
Lithuania	1	1	0
Italy	1	0	1
NI / Scotland	2	0	2
RoI / France	2	2	0
Germany / England	2	1	1
USA / England	1	1	0
NI / England	1	0	1
Scotland / RoI	1	1	0
Israel / France / Slovakia	1	1	0
USA / Wales	1	1	0
USA / NI	1	0	1
USA / RoI	1	1	0
Germany / NI	1	0	1
Netherlands / RoI / England	1	1	0
England / France	1	1	0
Poland / England	1	1	0
Germany/ RoI	1	1	0
Germany / France	1	0	1
France / Switzerland	1	1	0
England / Spain	1	1	0
RoI / England / Wales	1	1	0
RoI / England	1	1	0
**Total into Ireland**	1,309	428	881

Table S8. Originating countries and their frequencies of importations for Period A using maximum likelihood estimation.

Origin country	Both	Into RoI	Into NI
Republic of Ireland (RoI)	–	–	1
Northern Ireland (NI)	–	1	–
England	125	77	48
USA	22	14	8
Scotland	5	0	5
Netherlands	4	3	1
Japan	4	1	3
Germany	3	2	1
Spain	3	1	2
France	3	1	2
Wales	2	1	1
China	1	1	0
New Zealand	1	1	0
Bangladesh	1	1	0
Switzerland	1	1	0
Chile	1	0	1
Sweden	1	0	1
Belgium	1	1	0
**Total into Ireland**	178	105	73

Table S9. Originating countries and their frequencies of importations for Period B using maximum likelihood estimation.

Origin country	Both	Into RoI	Into NI
Republic of Ireland (RoI)	–	–	0
Northern Ireland (NI)	–	0	–
England	35	16	19
Scotland	3	2	1
Spain	1	1	0
**Total into Ireland**	39	19	20

Table S10. Originating countries and their frequencies of importations for Period C using maximum likelihood estimation.

Origin country	Both	Into RoI	Into NI
Republic of Ireland (RoI)	–	–	12
Northern Ireland (NI)	–	12	–
England	189	146	43
Germany	5	4	1
Poland	4	3	1
USA	4	4	0
Scotland	4	3	1
France	3	3	0
Sweden	3	3	0
Switzerland	2	2	0
Belgium	1	1	0
India	1	1	0
Iraq	1	1	0
Spain	1	1	0
Croatia	1	1	0
Italy	1	1	0
**Total into Ireland**	220	174	46

Table S11. Originating countries and their frequencies of importations for Period D using maximum likelihood estimation.

Origin country	Both	Into RoI	Into NI
Republic of Ireland (RoI)	–	–	39
Northern Ireland (NI)	–	28	–
England	490	279	211
Scotland	49	25	24
France	48	47	1
India	35	33	2
Germany	15	15	0
Spain	14	13	1
Netherlands	14	14	0
Denmark	12	11	1
Wales	12	5	7
USA	10	10	0
Sweden	8	8	0
Greece	4	4	0
Russia	4	4	0
Italy	3	3	0
Portugal	3	2	1
Belgium	2	2	0
Croatia	2	2	0
Nigeria	2	2	0
Hong Kong	1	1	0
Lithuania	1	1	0
Kenya	1	1	0
Australia	1	1	0
Turkey	1	1	0
South Africa	1	1	0
Uganda	1	1	0
Japan	1	1	0
Iceland	1	1	0
Zimbabwe	1	1	0
**Total into Ireland**	737	489	248

Table S12. Originating countries and their frequencies of importations for Period E using maximum likelihood estimation.

Origin country	Both	Into RoI	Into NI
Republic of Ireland (RoI)	–	–	31
Northern Ireland (NI)	–	27	–
England	1120	710	410
USA	518	392	126
France	106	96	10
Scotland	31	11	20
Germany	27	23	4
Spain	27	24	3
Canada	21	18	3
Poland	17	15	2
Brazil	9	9	0
Wales	7	3	4
India	7	4	3
Croatia	7	6	1
Denmark	5	5	0
South Africa	4	1	3
Belgium	4	4	0
Netherlands	4	4	0
Norway	3	3	0
Portugal	3	2	1
Japan	3	2	1
Australia	3	3	0
Turkey	2	2	0
Peru	2	2	0
Latvia	2	2	0
Romania	2	1	1
Italy	2	2	0
Switzerland	2	2	0
Bulgaria	1	0	1
Reunion	1	1	0
Mauritius	1	1	0
Finland	1	1	0
Austria	1	1	0
Indonesia	1	1	0
Slovenia	1	1	0
Seychelles	1	1	0
Czech Republic	1	1	0
Lithuania	1	1	0
**Total into Ireland**	1948	1355	593

Table S13. Originating countries and their frequencies of importations for Period F using maximum likelihood estimation.

Origin country	Both	Into RoI	Into NI
Republic of Ireland (RoI)	–	–	0
Northern Ireland (NI)	–	18	–
England	928	287	641
Denmark	137	39	98
Scotland	101	16	85
Germany	54	22	32
Switzerland	21	18	3
India	19	12	7
France	12	7	5
Sweden	12	6	6
USA	9	5	4
Wales	8	5	3
Netherlands	6	5	1
Norway	4	0	4
Poland	4	1	3
Israel	3	2	1
Spain	3	2	1
Slovakia	3	3	0
Lithuania	1	1	0
Australia	1	1	0
Italy	1	0	1
New Zealand	1	0	1
Austria	1	1	0
Belgium	1	1	0
Portugal	1	1	0
Hong Kong	1	0	1
Slovenia	1	1	0
Thailand	1	1	0
**Total into Ireland**	1334	437	897

Table S14. OLS linear regression statistics for estimating substitution rates of major imported lineages to Ireland.

**NI**	**N**	**slope**	**standard error**	R^2	p
Period A (Initial Introductions)	303	0.0438	0.0050	0.2047	1.078×10^{-16}
Period B (B.1.177)	285	0.0397	0.0022	0.5414	7.854×10^{-50}
Period C (B.1.1.7)	2409	0.0246	0.0012	0.1570	2.252×10^{-91}
Period D (Delta)	12025	0.0441	0.0006	0.3008	~ 0
Period E (Omicron (BA.1*))	186	0.0343	0.0045	0.2365	1.974×10^{-12}
Period F (Omicron (BA.2*))	2172	0.0391	0.0019	0.1574	8.293×10^{-83}
**RoI**	**N**	**slope**	**standard error**	R^2	p
Period A (Initial Introductions)	476	0.0539	0.0056	0.1616	6.536×10^{-20}
Period B (B.1.177)	594	0.0229	0.0018	0.2066	1.292×10^{-31}
Period C (B.1.1.7)	8895	0.0254	0.0008	0.1139	6.896×10^{-236}
Period D (Delta)	17968	0.0465	0.0004	0.4096	~ 0
Period E (Omicron (BA.1*))	1347	0.0255	0.0070	0.0099	2.476×10^{-4}
Period F (Omicron (BA.2*))	2612	0.0588	0.0018	0.2962	2.237×10^{-201}
**Ireland (NI + RoI)**	**N**	**slope**	**standard error**	R^2	p
Period A (Initial Introductions)	779	0.0439	0.0036	0.1637	4.803×10^{-32}
Period B (B.1.177)	879	0.0288	0.0014	0.3193	2.627×10^{-75}
Period C (B.1.1.7)	11304	0.0250	0.0006	0.1229	~ 0
Period D (Delta)	29993	0.0460	0.0003	0.3922	~ 0
Period E (Omicron (BA.1*))	1533	0.028886	0.005237	0.0195	4.064×10^{-8}
Period F (Omicron (BA.2*))	4784	0.0424	0.0011	0.2448	7.246×10^{-294}

References

1. O'Toole Á, Pybus OG, Abram ME, Kelly EJ, and Rambaut A. Pango lineage designation and assignment using SARS-CoV-2 spike gene nucleotide sequences. BMC Genomics 2022 Feb; 23:121. DOI: 10.1186/s12864-022-08358-2
2. Sanderson T. Taxonium: a web-based tool for exploring large phylogenetic trees. bioRxiv 2022 Jul :2022.06.03.494608. DOI: 10.1101/2022.06.03.494608
3. Jordahl K, Bossche JV den, Fleischmann M, McBride J, Wasserman J, Richards M, Badaracco AG, Gerard J, Snow AD, Tratner J, Perry M, Farmer C, Hjelle GA, Ward B, Cochran M, Taves M, Gillies S, Culbertson L, Bartos M, Caria G, Eubank N, sangarshanan, Flavin J, Rey S, maxalbert, Bilogur A, Ren C, Arribas-Bel D, Mesejo-León D, and Wasser L. geopandas/geopandas: v0.11.1. Zenodo, 2022 Jul. DOI: 10.5281/zenodo.6894736
4. Northern Ireland Statistics and Research Agency (NISRA). Census 2021 main statistics for Northern Ireland (phase 1). 2022. Available from: https://www.nisra.gov.uk/publications/census-2021-main-statistics-for-northern-ireland-phase-1 [Accessed on: 2023 Mar 10]
5. Central Statistics Office (CSO). Preliminary Actual and Percentage Change in Population 2016 - 2022. 2022. Available from: https://data.cso.ie/table/FP001 [Accessed on: 2022 Aug 18]
